# Normal circadian period length requires repression of *Npas2* by REV-ERB nuclear receptors

**DOI:** 10.1016/j.celrep.2025.116437

**Published:** 2025-10-14

**Authors:** Michael C. Tackenberg, Kristina M. Heliodoro, Lily C. Melink, Yifan Liu, Kun Zhu, Mitchell A. Lazar

**Affiliations:** 1 Institute for Diabetes, Obesity, and Metabolism, Perelman School of Medicine at the University of Pennsylvania, Philadelphia, PA 19104, USA; 2 Division of Endocrinology, Diabetes, and Metabolism, Department of Medicine, Perelman School of Medicine at the University of Pennsylvania, Philadelphia, PA 19104, USA; 3 Lead contact

## Abstract

REV-ERB nuclear receptors are integrated into the molecular circadian clock present in most mammalian cells. Loss of REV-ERBs (REV-ERB DKO) within the suprachiasmatic nucleus (SCN) *in vivo* leads to a marked shortening of the circadian period, but it remains unclear whether REV-ERB regulation of circadian period is tissue autonomous, if it is conserved across tissues, and how it is established. Here, we show that period shortening in the absence of REV-ERBs is tissue autonomous, is consistent between brain and liver, and is brought about through derepression of clock transcription factors NPAS2 and CLOCK. Thus, in addition to disruption of synchrony with the external environment, our results demonstrate that the circadian impacts of REV-ERB loss also include the alteration of core circadian properties with tissue-specific consequences.

## In brief

Loss of REV-ERB nuclear receptors shortens the free-running circadian period. Tackenberg et al. show that the period-shortening phenotype is conserved between the master circadian pacemaker, the SCN, and the peripheral clocks of the liver. The period-shortening phenotype requires the depression of *Npas2*, a REV-ERB target gene.

## Graphical abstract

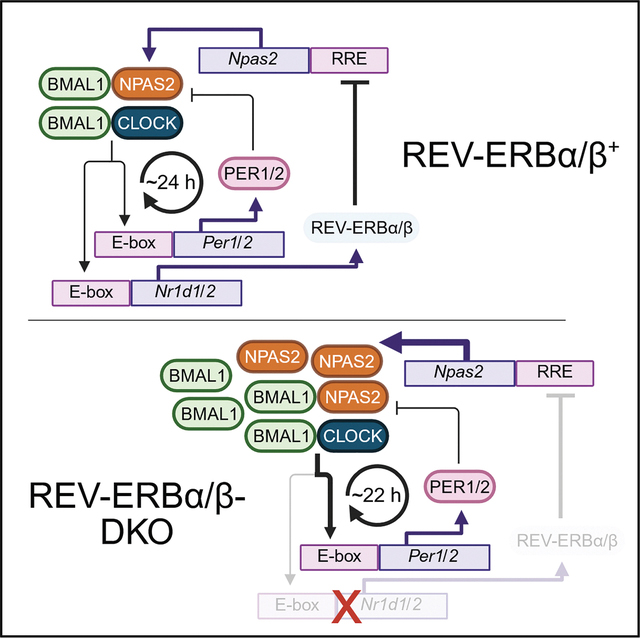

## INTRODUCTION

Circadian rhythms are generated in mammals by a self-sustaining transcription-translation feedback loop (TTFL).^1–3^ In each cycle of the TTFL, PER and CRY proteins accumulate in the cytosol following transcriptional activation by CLOCK (or NPAS2) and BMAL1 then re-enter the nucleus and suppress their own transcription through inhibition of CLOCK/NPAS2 and BMAL1 activity. REV-ERB nuclear receptors are major transcriptional repressors of multiple components of the positive arm of the TTFL, including *Arntl*, the gene encoding BMAL1,^4,5^ *Npas2*,^6^ and *Clock*.^7^ Whereas the deletion of both members of functionally overlapping clock gene pairs, such as *Per1* and *Per2*,^8^ *Cry1* and *Cry2*,^9^ or *Npas2* and *Clock*,^10^ or deletion of *Arntl* alone,^11^ leads to arrhythmicity in constant darkness, the loss of both REV-ERB isoforms, α and β (REV-ERB DKO) in the mammalian master clock, located in the suprachiasmatic nucleus (SCN), does not ablate rhythmicity but dramatically shortens the circadian period.^12^ Under normal 24-h light/dark (LD) cycles, this shortened period strains the relationship between the internal circadian clock and the external environment, leading to increased vulnerability to metabolic challenges.^12^

In addition to their function in the molecular clock, REV-ERBs have well-established roles in metabolism, acting via tissue-specific transcription factors to repress transcription of metabolic genes in organs such as the liver and adipose tissue.^13^ Thus, in conjunction with their role in the TTFL across cell types, REV-ERBs can perform multiple functions in metabolic tissues such as the liver, where deletion of REV-ERBs specifically within hepatocytes not only alters transcriptional rhythms in the liver but impacts transcriptional regulation of *de novo* lipogenesis,^14^ demonstrating a convergence of circadian and metabolic pathways. The disruption of REV-ERB function in either the SCN or the liver alone is therefore deleterious to metabolic health under standard 24-h light/dark conditions, but those negative impacts can be ameliorated through REV-ERB deletion in both tissues simultaneously^15^ or by limiting the conflicting signals between the SCN and liver through the ablation of the hepatic vagal afferent nerve.^16^

While previous work has focused on the impacts of REV-ERB loss on metabolic and transcriptomic outputs, our understanding of the consequences of REV-ERB deletion for the circadian system itself is limited to the period-shortening phenotype observed in the SCN and locomotor rhythms following knockout *in vivo*. Important questions remain regarding the tissue autonomy of the REV-ERB DKO period shortening, the conservation of that period shortening between tissues, and the mechanism by which they occur. To address these questions, we examined the circadian rhythms of isolated SCN and liver slices. We found that REV-ERB DKO shortens the SCN period in a tissue-autonomous fashion, an effect also observable in the REV-ERB DKO liver, through a mechanism involving direct repression of clock transcription factors CLOCK and NPAS2. These results demonstrate that REV-ERBs maintain the alignment of the clock period with that of the 24 h day via direct repression of core clock genes.

## RESULTS

### Maintenance of normal SCN period length by REV-ERBs is cell autonomous

**Figure 1.**
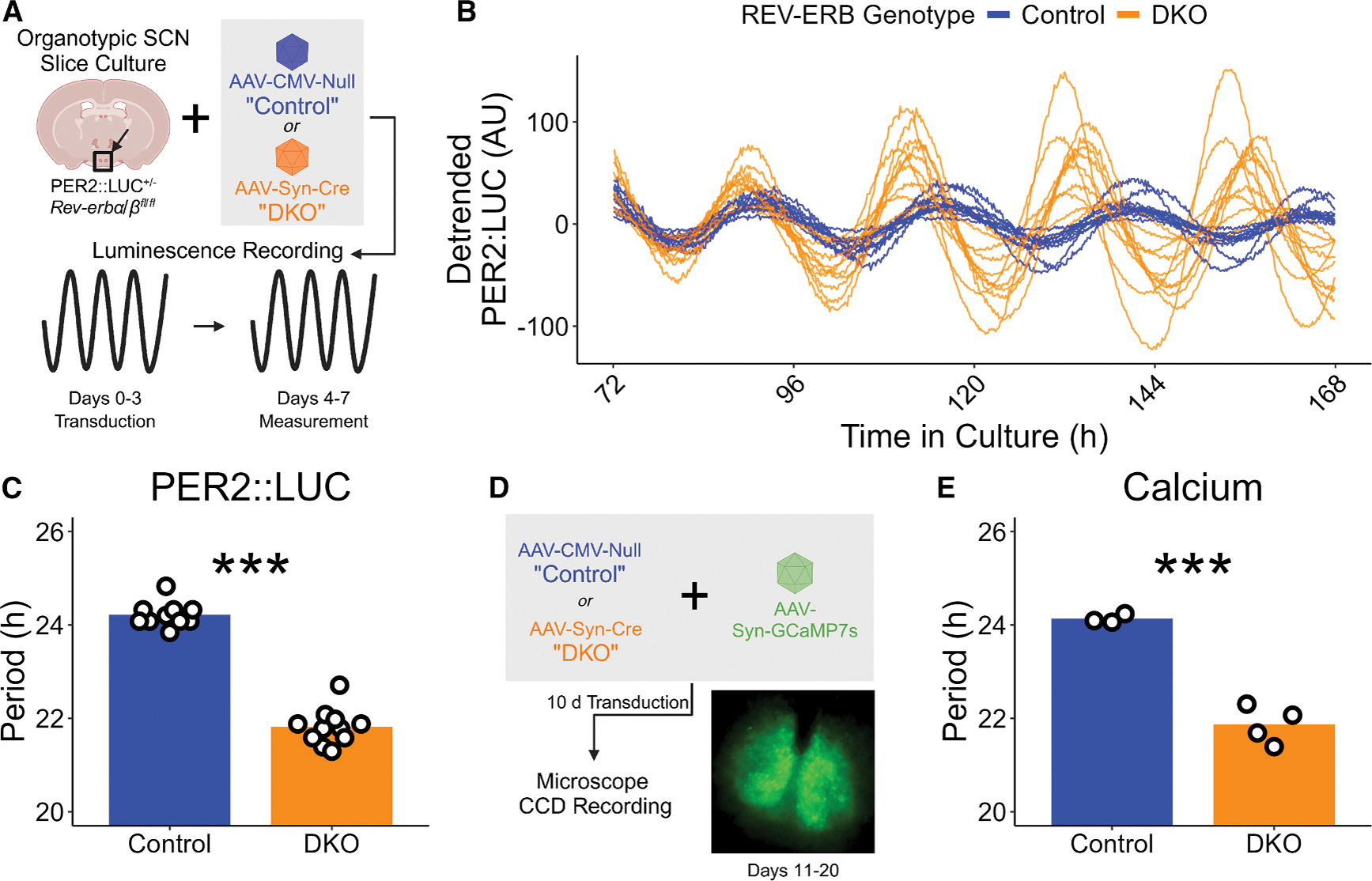
REV-ERB DKO shortens the period of the SCN PER2 rhythm tissue autonomously (A) Schematic view of the SCN organotypic slice preparation and PER2::LUC measurement. (B) Individual detrended PER2::LUC traces of control (blue) and REV-ERB DKO (orange) SCN slices. (C) Period length of the PER2::LUC rhythm for control (blue, mean ± SEM: 24.22 ± 0.083 h, *n* = 10; 5 male, 5 female) and REV-ERB DKO (orange, mean ± SEM: 21.81 ± 0.12 h, *n* = 11; 6 male, 5 female) SCN slices as measured by Lomb-Scargle periodogram on hours 72 through 168 of the recording. (*t* test, *p* < 0.0001). Bar height represents mean value. ***p < 0.001, **0.001 < *p* < 0.01, *0.01 < *p* < 0.05, ^ns^*p* > 0.05. (D) Schematic view of the SCN organotypic slice preparation and real-time bioluminescence imaging of GCaMP7s signal by CCD microscope. (E) Period length of GCaMP signal rhythms from control (blue, mean ± SEM: 24.13 ± 0.055 h, *n* = 3; all female) and REV-ERB DKO (orange, 21.86 ± 0.20 h, *n* = 4, all female) SCN slices as measured by Lomb-Scargle periodogram on hours 0 through 240 of the recording, which began on the 9^th^ day following slicing (*t* test, *p* = 0.0002). Bar height represents mean value. ***p < 0.001, **0.001 < *p* < 0.01, *0.01 < *p* < 0.05, ^ns^*p* > 0.05.

To determine whether the generation of the period-shortening phenotype of REV-ERB DKO in the SCN is tissue autonomous, we made organotypic SCN slices from adult REV-ERBα and β floxed mice expressing the PER2::LUCIFERASE (PER2::LUC) fusion reporter and initiated REV-ERB DKO entirely *ex vivo*. We compared the rhythms of *ex vivo* SCN slices immediately treated topically with either AAV-CMV-Null (“control”) or AAV-Syn-Cre (“DKO”), followed by 72 h for viral transduction (Figure 1A). For 4 days following transduction, the PER2::LUC signal was measured by luminometer every 10 min (Figure 1B). REV-ERB DKO in the SCN *ex vivo* shortened the period by approximately 2.5 h (Figure 1C), consistent with previous results culturing the SCN following REV-ERB deletion *in vivo*,^12^ demonstrating that the period-shortening phenotype is a tissue-autonomous effect of REV-ERB knockout rather than a network-driven after-effect of knockout *in vivo*. Similar period shortening was also observed in both male and female mice (Figure S1). To ensure that the period-shortening phenotype was not an artifact of the PER2::LUC reporter system, we assessed the REV-ERB DKO period length using a second reporter of SCN period: calcium rhythms. We made organotypic SCN slices from REV-ERBα and β floxed mice (with wild-type PER2, rather than the PER2::LUC reporter), and upon culture treated with either AAV-CMV-Null (“control”) or AAV-Syn-Cre (“DKO”) along with AAV encoding the calcium reporter GCaMP7s (AAV-Syn-GCaMP7s). Following 10 days for transduction, we imaged the SCN slices using a charge-coupled device (CCD) microscope (Figure 1D). Consistent with PER2::LUC measurements, the period of the REV-ERB DKO GCaMP7s rhythm was shortened compared to controls (Figure 1E).

### REV-ERBs also control normal circadian period length in the liver

**Figure 2.**
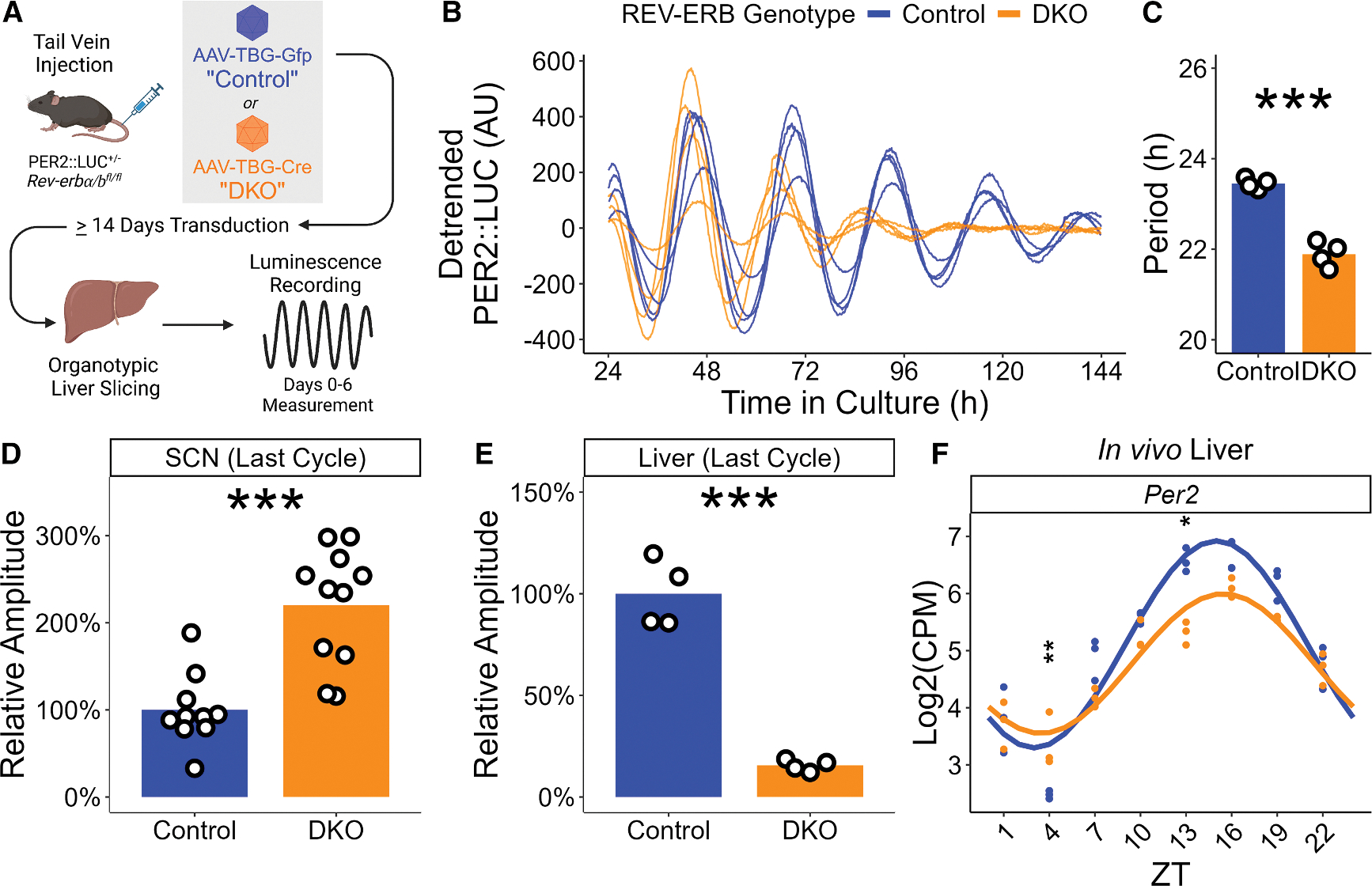
REV-ERB DKO shortens the period of the liver PER2 rhythm (A) Schematic view of the tail vein injection, liver organotypic slice preparation, and PER2::LUC recording. (B) Individual detrended PER2::LUC traces of control (blue) and REV-ERB DKO (orange) liver slices. (C) Period length of the PER2::LUC rhythm for control (blue, mean ± SEM: 23.45 ± 0.06 h, *n* = 4, all male) and REV-ERB DKO (orange, mean ± SEM: 21.89 ± 0.14 h, *n* = 4, all male) liver slices as measured by Lomb-Scargle periodogram on hours 24 through 144 of the recording (*t* test, *p* < 0.0001). Bar height represents mean value. ***p < 0.001, **0.001 < *p* < 0.01, *0.01 < *p* < 0.05, ^ns^*p* > 0.05. (D) PER2::LUC amplitude of control (blue, mean ± SEM: 100% ± 13%, *n* = 10) and REV-ERB DKO (orange, mean ± SEM: 220% ± 20%, *n* = 11) SCN slices on the final cycle, relative to control mean (*t* test, *p* = 0.0001). Bar height represents mean value. ***p < 0.001, **0.001 < *p* < 0.01, *0.01 < *p* < 0.05, ^ns^*p* > 0.05. (E) PER2::LUC amplitude of control (blue, mean ± SEM: 100% ± 8%, *n* = 4; all male) and REV-ERB DKO (orange, mean ± SEM: 15% ± 1%, *n* = 4; all male) liver slices on the final cycle, relative to control mean (*t* test, *p* < 0.0001). Bar height represents mean value. ***p < 0.001, **0.001 < *p* < 0.01, *0.01 < *p* < 0.05, ^ns^*p* > 0.05. (F) *Per2* gene expression for control (blue) and HepDKO (orange) livers as measured by RNA-seq on livers collected from mice on a 12:12 LD cycle from Guan et al.^14^ Dots represent the log_2_(CPM), and lines represent a linear model fit of a 24-h-period linear-form sine + cosine. Statistics performed on log_2_(CPM) values; two-way ANOVA with Šidák’s multiple comparisons test: *p*(control – DKO, ZT 4) = 0.008, *p*(control – DKO, ZT 13) = 0.0001. ***p < 0.001, **0.001 < *p* < 0.01, *0.01 < *p* < 0.05, ^ns^*p* > 0.05.

Because REV-ERBs act within the TTFL found in most cells throughout the mammalian body, we hypothesized that period shortening upon REV-ERB deletion would also occur in peripheral tissues. Liver slices were made from adult REV-ERBα and β floxed mice heterozygous for the PER2::LUC reporter that had been injected at least 14 days prior via the tail vein with AAV-TBG-Gfp (“control”) or AAV-TBG-Cre (“DKO”; Figure 2A). PER2::LUC expression was measured every 10 minutes by luminometer for 5 days immediately upon culture (Figure 2B). Consistent with its effects in the SCN, REV-ERB DKO shortened the period of liver slices by approximately 1.5 h (Figure 2C), indicating that the role of REV-ERBs in maintaining normal circadian periods extends to peripheral clocks.

### Tissue-specific effects of loss of REV-ERBs on the molecular clock

While deletion of REV-ERBs shortened the period similarly in SCN and liver, we noted diametrically opposite effects on circadian peak-trough amplitude, which was increased in SCN (Figure 2D) but markedly decreased in the liver (Figure 2E). Importantly, the differences in amplitude between each of the REV-ERB DKO slices of each tissue and their respective controls was not due to changes in survival during the recording window, as overall luminescence levels of all slices remained elevated throughout (Figures S2A and S2B). Further, while the amplitude of the REV-ERB DKO liver slice PER2::LUC rhythm diminished over time, it remained greater than 8 times the typical noise level of the recording throughout the analyzed recording window of all slices (Figures S2C and S2D), indicative of meaningful rhythmicity even when damped. To determine if the amplitude difference observed *ex vivo* is relevant to the liver *in vivo*, we analyzed existing RNA sequencing (RNA-seq) data from control and hepatocyte-specific REV-ERB DKO livers collected in 12:12 LD^14^ and found that the peak expression of *Per2* was significantly decreased in REV-ERB DKO as compared to controls (Figure 2F). These data show that, while the control of period length by REV-ERBs occurs in central and peripheral clocks, loss of REV-ERB has tissue-specific consequences for the amplitude of the PER2::LUC rhythm.

**Figure 3.**
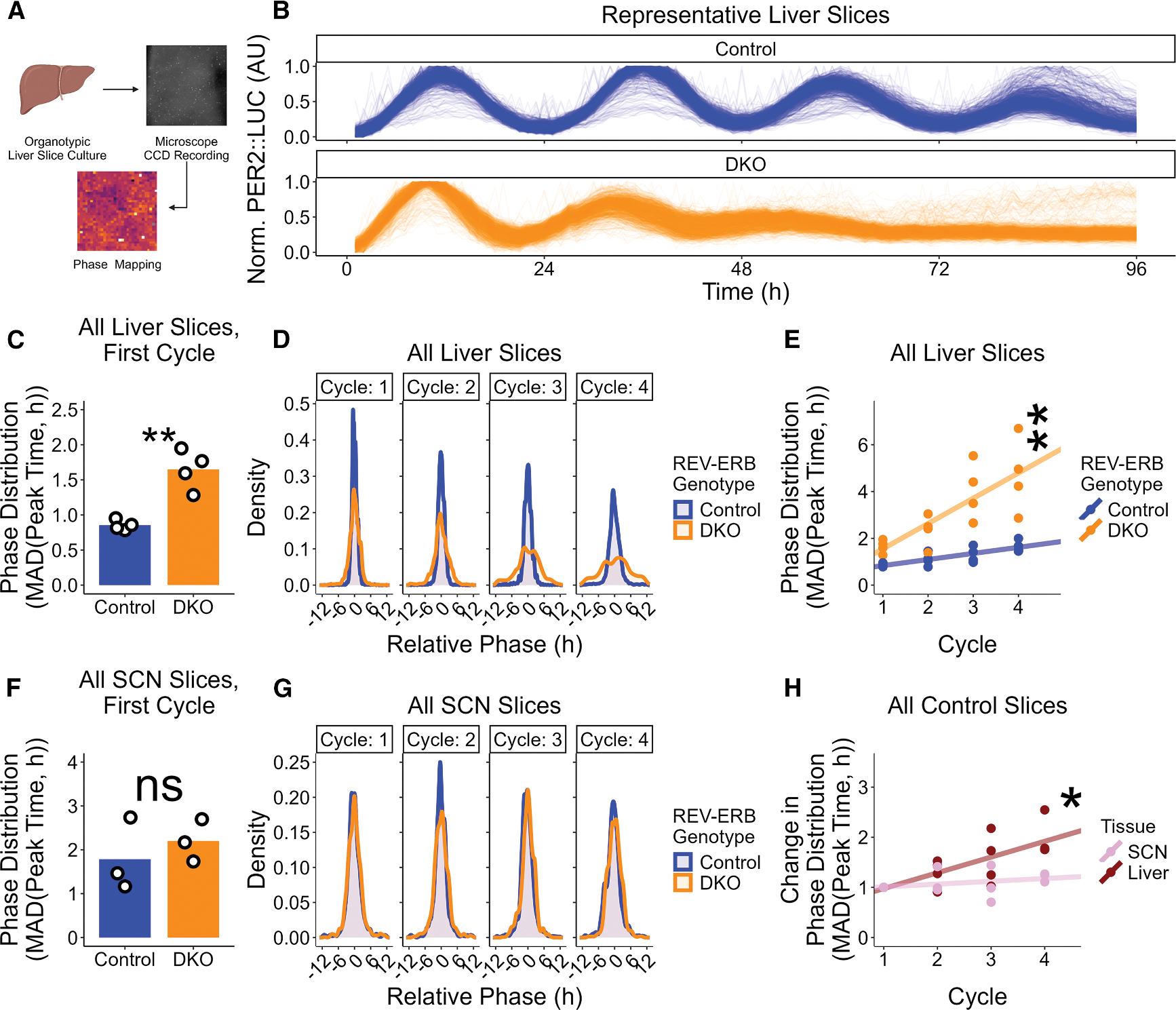
REV-ERB DKO has tissue-specific effects on PER2::LUC amplitude explained by liver phase coherence (A) Schematic view of the real-time bioluminescence recording of PER2::LUC signal by CCD microscopy. Microscope recording began on the 2^nd^ day after slicing. (B) Individual PER2::LUC traces from pixels from a representative control (top, blue) and REV-ERB DKO (bottom, orange) liver slice. (C) Phase distribution as measured by median absolute deviation (MAD) of relative peak time values for control (blue, mean ± SEM: 0.852 ± 0.037 h, *n* = 4; 2 male, 2 female) and REV-ERB DKO (orange, mean ± SEM: 1.648 ± 0.146 h, *n* = 4; 2 male, 2 female) liver slices (*t* test, *p* = 0.0016). Bar height represents mean value. ***p < 0.001, **0.001 < *p* < 0.01, *0.01 < *p* < 0.05, ^ns^*p* > 0.05. (D) Frequency distribution of relative peak times of all pixels in all control (blue) and REV-ERB DKO (orange) liver slices for the first four cycles recorded. (E) Phase distribution as measured by median absolute deviation (MAD) of relative peak time values for control (blue) and REV-ERB DKO (orange) liver slices (extra sum of squares F test for unshared slope, *p* = 0.0016). Line represents linear fit of the data. ***p < 0.001, **0.001 < *p* < 0.01, *0.01 < *p* < 0.05, ^ns^*p* > 0.05. (F) Phase distribution as measured by median absolute deviation (MAD) of relative peak time values for control (blue, mean ± SEM: 1.789 ± 0.835 h, *n* = 3, 1 male, 2 female) and REV-ERB DKO (orange, mean ± SEM: 2.200 ± 0.278 h, *n* = 3, 2 male, 1 female) SCN slices (*t* test, *p* = 0.5008). Bar heights represent mean values. ***p < 0.001, **0.001 < *p* < 0.01, *0.01 < *p* < 0.05, ^ns^*p* > 0.05. (G) Frequency distributions of relative peak times of all pixels in all control (blue) and REV-ERB DKO (orange) SCN slices over the first four cycles recorded. (H) Change in phase distribution relative to cycle 1 as measured by median absolute deviation (MAD) of relative peak time values for control SCN (pink) and control liver (dark red) slices (extra sum of squares F test for unshared slope, *p* = 0.0138). Line represents linear fit of the data. ***p < 0.001, **0.001 < *p* < 0.01, *0.01 < *p* < 0.05, ^ns^*p* > 0.05.

Since the amplitude of the PER2::LUC rhythm in the REV-ERB DKO liver was initially comparable with that of control liver, we considered whether the damping was the result of progressive desynchronization in the liver but not in the SCN. To test this hypothesis, we imaged organotypic liver slices from control and REV-ERB DKO mice using a CCD microscope (Figure 3A), followed by analysis of 4 days of the PER2::LUC signal from each pixel (Figure 3B). In all slices, >97% of pixels remained significantly rhythmic as measured by Lomb-Scargle periodogram (α = 0.01). Remarkably, the phase distribution was widened in REV-ERB DKO liver slices on the first cycle in the microscope (Figure 3C), and the effect on REV-ERB DKO phase distribution grew more pronounced with time (Figure 3D, quantified in Figure 3E). Thus the peak times of the PER2::LUC signal across the liver were more dispersed in the REV-ERB DKO liver slices than in control slices. By contrast, the same analysis in SCN slices (Figures S3A and S3B) revealed that median absolute deviation of PER2:::LUC peak times was not increased in REV-ERB DKO SCN slices compared to controls (Figure 3F) and that phase distribution of the control and REV-ERB DKO SCN largely overlapped (Figure 3G, quantified in Figure S3C). The phase distribution difference was therefore unique to the liver and thus likely to account for the liver-specific damping. Comparison of the change in phase distribution over time in control SCN and control liver slices revealed that the liver normally becomes more phase distributed over time than the tightly networked control SCN (Figure 3H). These results demonstrate that the inherent circadian properties of a given tissue create tissue-specific vulnerabilities to consistent circadian changes by REV-ERB loss, with the more weakly coupled liver being more prone to destabilization upon REV-ERB DKO than the more well-coupled SCN.

### Direct repression of clock gene Npas2 by REV-ERBs is required to maintain the nearly 24 h period of the normal clock

To understand the mechanism of period control by REV-ERBs, we initially focused on Casein kinase 1ε (CK1ε), which alters the degradation rate of PER proteins to modify circadian period length.^17–19^ However, while treatment with the CK1δ/ε inhibitor PF670462^20^ (Figure S4A) led to period lengthening of control SCN slices, it similarly lengthened the period of REV-ERB DKO SCN slices, which remained nearly 3 h shorter (Figures S4B and S4C), suggesting that the period shortening by the REV-ERB DKO involves an alternative mechanism. Indeed, following treatment with the protein synthesis inhibitor cycloheximide (CHX) following REV-ERB DKO induction (Figures S5A and S5B), the half-life of PER2 was indistinguishable between control and REV-ERB DKO SCN (Figures S5C and S5D), demonstrating that the REV-ERB DKO period shortening is not mediated by the reduction in PER2 stability commonly seen in other period-shortening mutations.^19^

**Figure 4.**
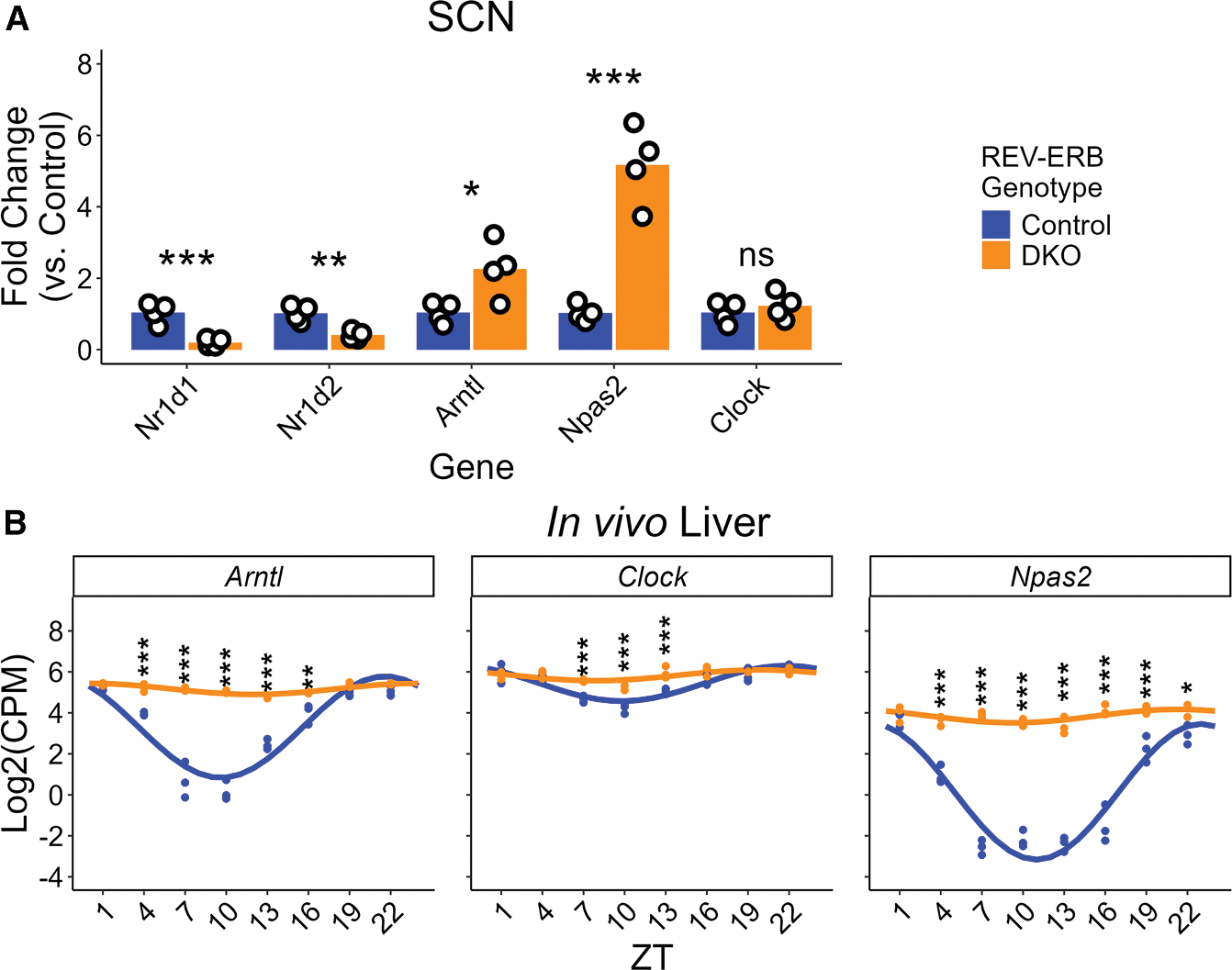
Loss of REV-ERBs leads to upregulation of target genes in SCN and liver (A) Gene expression change for REV-ERB DKO (orange, *n* = 4; 1 male, 3 female) SCN slices relative to control (blue, *n* = 4; 2 male, 2 female) slices (control slice gene expression = 1) at approximately the peak of PER2::LUC expression (CT 12). Statistics performed on ΔCt (versus housekeeping gene *Rpl19*) values for each gene: *Nr1d1* (control: 3.215 ± 0.225; DKO: 5.731 ± 0.445), *Nr1d2* (control: 2.611 ± 0.166, DKO: 3.951 ± 0.215), *Arntl* (control: 3.203 ± 0.219, DKO: 2.101 ± 0.278), *Npas2* (control: 8.163 ± 0.166, DKO: 5.820 ± 0.163), and *Clock* (control: 3.933 ± 0.228, DKO: 3.697 ± 0.225). Two-way ANOVA, *p* (gene) < 0.0001, *p*(REV-ERB genotype) = 0.8251, *p*(interaction) < 0.0001. Šidák’s multiple comparisons test (control – DKO): *p*(*Nr1d1*) < 0.0001, *p* (*Nr1d2*) = 0.0028, *p*(*Arntl*) = 0.0173, *p* (*Npas2*) < 0.0001, *p*(*Clock*) = 0.9694. Values graphed are 2^(−ΔΔCt)^ fold change values (versus control slice mean). Bar height represents mean values. ***p < 0.001, **0.001 < *p* < 0.01, *0.01 < *p* < 0.05, ^ns^*p* > 0.05. (B) *Arntl*, *Npas2*, and *Clock* gene expression for control (blue) and HepDKO (orange) livers as measured by RNA-seq on livers collected from mice on a 12:12 LD cycle from Guan et al.^14^ Dots represent the log_2_(CPM) and lines represent a fit of a 24-h-period linear-form sine + cosine linear model. Statistics performed on log_2_(CPM) values; two-way ANOVA (main effects of ZT and REV-ERB genotype for each gene). For all three genes, *p*(ZT) < 0.0001 and *p*(REV-ERB Genotype) < 0.0001. Šidák’s multiple comparisons test (control – DKO at each ZT): significant differences detected at ZT 4 (*p* = 0.0001), 7 (*p* < 0.0001), 10 (*p* < 0.0001), 13 (*p* < 0.0001), and 16 (*p* = 0.003) for *Arntl*; ZT 7 (*p* = 0.0007), 10 (*p* < 0.0001), and 13 (*p* = 0.0009) for *Clock*; and ZT 4 (*p* < 0.0001), 7 (*p* < 0.0001), 10 *(p* < 0.0001), 13 (*p* < 0.0001), 16 (*p* < 0.0001), 19 (*p* < 0.0001), and 22 (*p* = 0.0185) for *Npas2*. ***p < 0.001, **0.001 < *p* < 0.01, *0.01 < *p* < 0.05, ^ns^*p* > 0.05.

We next hypothesized that the circadian effects of REV-ERB DKO was established through direct transcriptional derepression of REV-ERB target genes. Because we observed increased amplitude of PER2::LUC in the REV-ERB DKO SCN (and in the early cycles of REV-ERB DKO liver), we focused on *Arntl* (coding for BMAL1) and *Npas2* (heterodimer partner of BMAL1), core clock genes that are known REV-ERB targets^4–6^ and whose protein products are transcriptional activators of *Per2*.^4^ As expected, expression of these genes at circadian time (CT) 12 (calculated by the expression of PER2::LUC in each slice to ensure phase-matching between samples) in control and REV-ERB DKO SCN slices following induction of the period-shortening phenotype (Figure S6) were markedly increased, especially *Npas2* (Figure 4A). Similar results were observed in the liver, with *Arntl*, *Npas2*, and *Clock* all significantly upregulated in hepatocyte-specific REV-ERB DKO livers *in vivo* (Figure 4B, re-analyzed from Guan et al.^14^). Though *Arntl* is a canonical circadian target for REV-ERB-mediated transcriptional repression, we found that its overexpression in SCN slices did not shorten period length even with combined overexpression of *Npas2* (Figures S7A and S7B). Further, treatment of REV-ERB DKO SCN slices with AAV-mediated short hairpin RNA (shRNA) targeting *Arntl* (Figures S8A and S8B) caused reduction in rhythmic power that interfered with meaningful period length measurements (Figures S8C and S8D) and precluded amplitude measurement. Note that although in this experiment we observed an unusually short period in the shCtrl-treated control group (23.12 ± 0.61 h), the shCtrl-treated DKO slices exhibited the expected period shortening (22.16 ± 0.39 h) consistent with other experiments.

**Figure 5.**
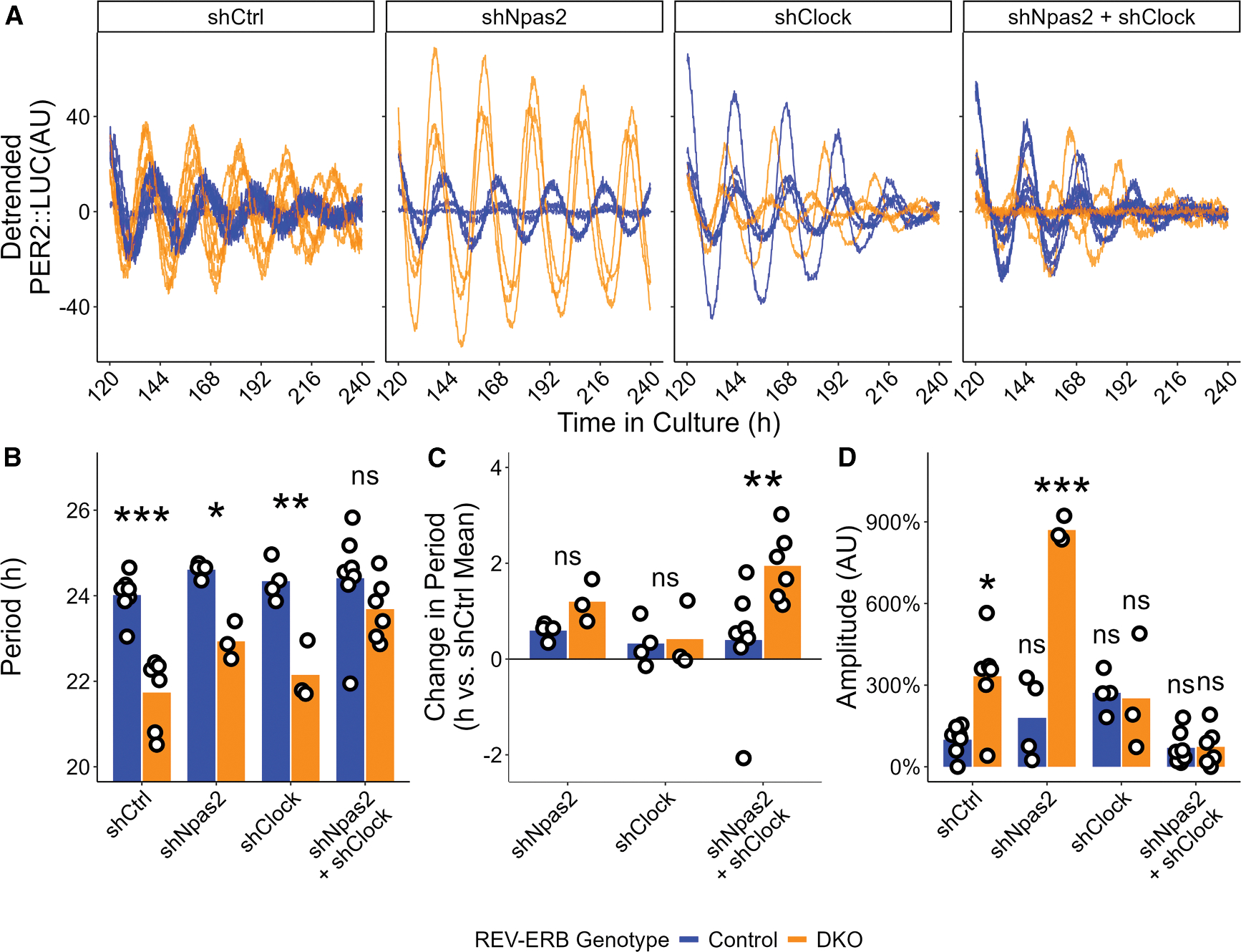
Simultaneous knockdown of *Npas2* and *Clock* restores control period and amplitude to the REV-ERB DKO SCN (A) Individual detrended PER2::LUC traces of control (blue) and REV-ERB DKO (orange) SCN slices for shCtrl-, shNpas2-, shClock-, and shNpas2 + shClock-treated SCN slices. (B) Period length of the PER2::LUC rhythm for control (blue) and REV-ERB DKO (orange) SCN slices treated with shCtrl (control mean ± SEM: 24.02 ± 0.19 h, *n* = 7, 4 male, 3 female; DKO mean ± SEM: 21.74 ± 0.35 h, *n* = 6, 2 male, 4 female), shClock (control mean ± SEM: 24.34 ± 0.23 h, *n* = 4, 2 male, 2 female; DKO mean ± SEM: 22.15 ± 0.40 h, *n* = 3, 1 male, 2 female), shNpas2 (control mean ± SEM: 24.61 ± 0.09 h, *n* = 4, 2 male, 2 female; DKO mean ± SEM: 22.93 ± 0.26 h, *n* = 3, 1 male, 2 female), or shNpas2 + shClock (control mean ± SEM: 24.41 ± 0.46 h, *n* = 7, 2 male, 2 female; DKO mean ± SEM: 23.68 ± 0.29 h, *n* = 6, 2 male, 4 female) as measured by Lomb-Scargle periodogram on hours 120 through 240 of the recording (two-way ANOVA: *p*(shRNA treatment) = 0.0032, *p*(REV-ERB genotype) < 0.0001, *p*(interaction) = 0.0683; Šidák’s multiple comparisons test: *p*(shCtrl, control – DKO) < 0.0001, *p*(shClock, control – DKO) = 0.0027, *p*(shNpas2, control – DKO) = 0.027), *p*(shNpas2 + shClock, control – DKO) = 0.3320). Bar height represents mean values. ***p < 0.001, **0.001 < *p* < 0.01, *0.01 < *p* < 0.05, ns *p* > 0.05. (C) Change in period length, relative to shCtrl-treated mean for each group, of control (blue) and REV-ERB DKO (orange) SCN slices treated with shClock (control mean ± SEM: 0.32 ± 0.23 h; DKO mean ± SEM: 0.41 ± 0.40 h), shNpas2 (control mean ± SEM: 0.59 ± 0.09 h; DKO mean ± SEM: 1.20 ± 0.26 h), or shNpas2 + shClock (control mean ± SEM: 0.39 ± 0.46 h; DKO mean ± SEM: 1.95 ± 0.29 h; two-way ANOVA: *p*[shRNA treatment] = 0.1293, *p*[REV-ERB genotype] = 0.0307, *p* [interaction] = 0.1520; Šidák’s multiple comparisons test: *p*[shClock, control – DKO] = 0.9983, *p*[shNpas2, control – DKO] = 0.7034, *p*[shNpas2 + shClock, control – DKO] = 0.0065). Bar height represents mean values. ***p < 0.001, **0.001 < *p* < 0.01, *0.01 < *p* < 0.05, ^ns^*p* > 0.05. (D) PER2::LUC amplitude, relative to shCtrl-treated controls, on the final cycle of control (blue) and REV-ERB DKO (orange) SCN slices treated with shCtrl (control mean ± SEM: 100% ± 20%, *n* = 7, 4 male, 3 female; DKO mean ± SEM: 333% ± 69%, *n* = 6, 2 male, 4 female), shNpas2 (control mean ± SEM: 179% ± 76%, *n* = 4, 2 male, 2 female; DKO mean ± SEM: 869% ± 27%, *n* = 3, 1 male, 2 female), shClock (control mean ± SEM: 272% ± 37%, *n* = 4, 2 male, 2 female; DKO mean ± SEM: 251% ± 124%, *n* = 3, 1 male, 2 female), or shNpas2 + shClock (control mean ± SEM: 70% ± 23%, *n* = 7, 4 male, 3 female; DKO mean ± SEM: 73% ± 29%, *n* = 6, 2 male, 4 female). Amplitudes of each REV-ERB genotype/shRNA treatment combination were compared to shCtrl-treated controls using Dunnett’s test with Šidák’s correction (*p*[DKO + shCtrl] = 0.02829, *p*[control + shNpas2] = 0.9999, *p*[DKO + shNpas2] < 0.0001), *p*(control + shClock) = 0.5274, *p*(DKO + shClock) = 0.8862, *p*(control + shNpas2 + shClock) > 0.9999, *p*(DKO + shNpas2 + shClock) = 0.9999. Bar height represents mean value. ***p < 0.001, **0.001 < *p* < 0.01, *0.01 < *p* < 0.05, ^ns^*p* > 0.05.

**Figure 6.**
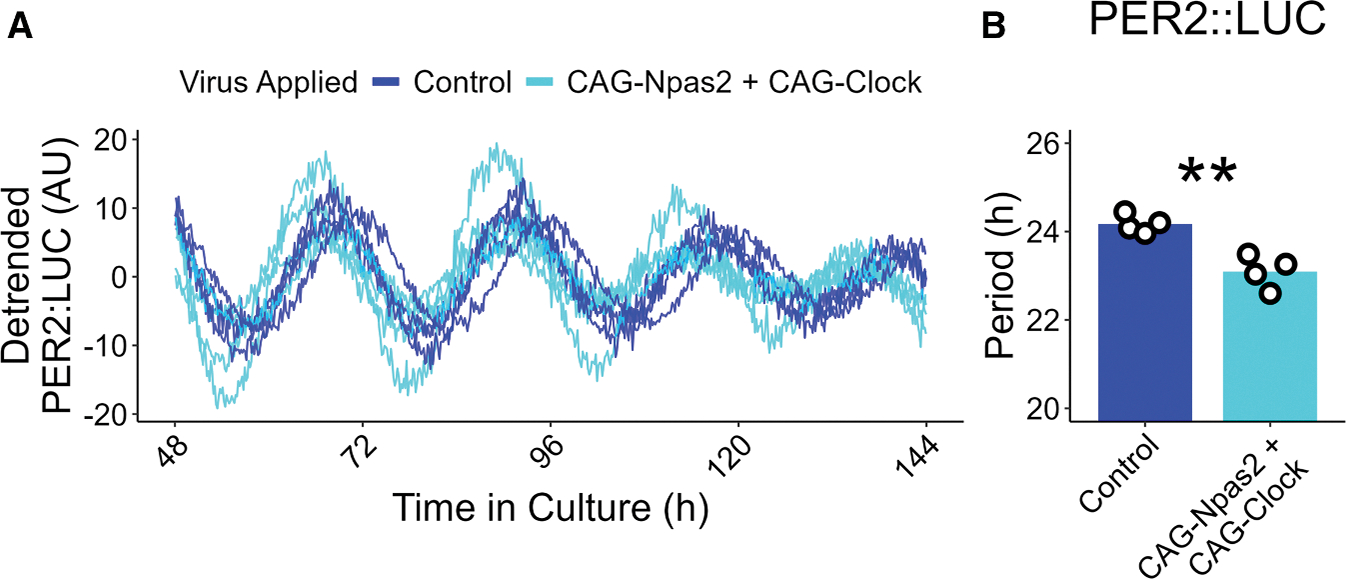
Overexpression of *Npas2* and *Clock* shortens the period of SCN slices (A) Individual detrended PER2::LUC traces of control (blue) and CAG-Npas2 + CAG-Clock-treated (light blue) SCN slices. (B) Period length of the PER2::LUC rhythm for control (blue, mean ± SEM: 24.17 ± 0.10 h, *n* = 4, 2 male, 2 female) and CAG-Npas2 + CAG-Clock-treated (light blue, mean ± SEM: 23.10 ± 0.19 h, *n* = 4, 2 male, 2 female) SCN slices as measured by Lomb-Scargle periodogram (*t* test, *p* = 0.0025). Bar height represents mean value. ***p < 0.001, **0.001 < *p* < 0.01, *0.01 < *p* < 0.05, ^ns^*p* > 0.05.

*Npas2* was upregulated more substantially in the REV-ERB DKO SCN than *Arntl*, and because it is a direct and robust repression target of REV-ERBs,^6,14,21^ we hypothesized that repression of *Npas2* by REV-ERB was required to maintain the near-24 h period length of the SCN. Depletion of *Npas2* alone did not have much effect on the period length (Figures S9A and S9B), and neither did depletion of *Clock* (Figures S9C, S9D, 5A, and 5B). Nevertheless, because NPAS2 and CLOCK have redundant functions in the TTFL mechanism,^10,22,23^ we considered whether REV-ERBs maintained normal period length by controlling the total amount of BMAL1 heterodimer partner. Remarkably, treatment of DKO SCN slices with shRNA targeting both *Npas2* and *Clock* rescued the shortened period phenotype (Figures 5A and 5B). Importantly, the same treatment has little effect on the normal period length of control SCN slices. Relative to shCtrl-treated slices of the same REV-ERB genotype, REV-ERB DKO SCN slices depleted of NPAS2 and CLOCK exhibited a period lengthening of approximately 2 h that was not observed in control SCN slices (Figure 5C). Interestingly, treatment with shClock alone and shNpas2 + shClock combined restored control-like amplitude to the REV-ERB DKO slices, while shNpas2 treatment alone exacerbated the amplitude increase of the DKO slices (Figure 5D). As further evidence of the role of *Npas2* and *Clock* on regulating period length, we found that SCN slices treated simultaneously with CAG-Npas2 and CAG-Clock viruses had shortened free running period compared with controls (Figures 6A and 6B), though the treatment had no effect on amplitude (Figure S10). Thus, the repression of NPAS2 by REV-ERBs is necessary for maintenance of normal circadian period length, and their overexpression is sufficient to shorten period.

## DISCUSSION

Our results demonstrate that period regulation by REV-ERBs is tissue autonomous, consistent between the SCN and liver, and driven by derepression of the transcription factors *Npas2* and *Clock*. These findings reveal that the role of REV-ERBs in the TTFL is more substantial than solely regulating *Arntl*/BMAL1 and that the physiological impacts of the loss of REV-ERBs go beyond the induction of a mismatch in period length between the endogenous circadian rhythm of an organism and the cycling of its environment, but rather involve distinct, tissue-specific circadian vulnerabilities such as reduced network coherence in the weakly coupled liver.

Period regulation in the circadian system is brought about through cell-autonomous changes to the TTFL itself^18,24–26^ or by network changes to the SCN following environmental exposure (referred to as after-effects).^27–29^ Previous experiments in which REV-ERB DKO was induced in the SCN *in vivo* followed by measurements both *in vivo* and *ex vivo* therefore left open the possibility that period shortening observed in locomotor rhythms in constant darkness *in vivo* or in PER2::LUC rhythms *ex vivo* could be the result of an after-effect of impaired or altered entrainment to the light/dark cycle. Here, we examined the REV-ERB DKO condition in the SCN with knockout induced entirely *ex vivo* and thus lacking any connections between the SCN and other tissues, and as such with no means of entrainment, conclusively showing that period regulation by REV-ERBs occurs in a stable, tissue-autonomous fashion.

While the progression of the TTFL is largely common across cells and tissues, the many tissue-specific roles of REV-ERBs raises the possibility that period, amplitude, or phase response regulation by REV-ERBs may also be tissue specific. Though limitations in the lifespan of organotypically cultured liver slices prevented us from initiating REV-ERB DKO entirely *ex vivo* in the liver as we had done in the SCN, we found that the circadian impacts of REV-ERB deletion were remarkably consistent between SCN and liver, with both tissues having a shortened period in the REV-ERB DKO condition. One important difference, however, was the apparent amplitude of the SCN and the liver. In the SCN, the amplitude of the PER2 rhythm was substantially upregulated by REV-ERB DKO, while in the liver, amplitude of the REV-ERB DKO slices was initially matched with controls but rapidly declined over time. Real-time bioluminescence microscopy of PER2::LUC revealed that, unlike in the SCN, REV-ERB DKO liver phase distribution is increased relative to controls, suggesting that the apparent reduction in amplitude is in fact the result of a loss of coherence in the REV-ERB DKO liver. Overall, these data indicate that while direct circadian impacts of REV-ERB DKO are conserved across tissues, the network coherence of each tissue renders well-coupled tissues like the SCN less vulnerable to certain effects of the DKO than other, more loosely coupled tissues like the liver.

Because the SCN and liver are part of an interconnected circadian network *in vivo*, our experiments focused on these two tissues in isolation to examine their endogenous properties *ex vivo* in the REV-ERB DKO condition. In the intact animal, SCN signaling to the liver is critical, and even mice lacking a functional clock in the liver are able to maintain rhythmicity of gene expression through cues from the intact SCN.^30,31^ Unlike the clock-less liver, however, our results indicate that the REV-ERB DKO liver is actually clock-competent, but with a highly unusual period length. As such, signaling from the SCN to the liver will be met not with a proper 24-h circadian clock nor the blank canvas of a non-functional clock, but with an improperly timed clock. By measuring these features *ex vivo*, we have revealed new information about how REV-ERB DKO specifically within the liver causes metabolic dysfunction.^14,32^

In addition to providing insights into how the REV-ERB DKO liver clock will participate in the circadian network between tissues, our results also shed light on how the intracellular synchrony of the *in vivo* REV-ERB DKO liver may be impacted. Under normal conditions, the liver clock is strongly entrained to signals from the SCN and feeding, but coherent rhythmicity persists even when the SCN is ablated,^33^ indicating a sufficiently strong intratissue coupling within the liver. Albeit weaker, the intratissue coupling within the liver was apparent as coherent rhythms persisted for a week or more in slices cultured *ex vivo* in the absence of SCN signaling. This coupling in liver slices was weakened in the absence of REV-ERBs, and this change translated to the liver *in vivo* where peak expression of *Per2* in was decreased in hepatocyte-specific REV-ERB DKO.

Although changes in stability of PER2 has been shown to cause period shortening, our experiments rule out this mechanism of period shortening in the REV-ERB DKO SCN. Rather, the direct repression of *Npas2*, a core clock gene whose protein product is a heterodimer partner of BMAL1 in the core clock, is required for REV-ERBs to maintain the normal clock period. NPAS2 and CLOCK have similar roles in the clock,^10,22^ and our data show that REV-ERB DKO-induced upregulation of NPAS2 is only sufficient to cause period shortening in the presence of at least normal levels of CLOCK, suggesting that it is the total amount of the two BMAL1 heterodimer partners (NPAS2 and CLOCK) that control the normal period. In SCN, this appears to be primarily due to the large derepression of *Npas2*, since *Clock* gene expression was only minimally affected in the REV-ERB DKO SCN. However, in liver, the loss of REV-ERBs significantly induces *Clock* gene expression (Figure 4B),^14^ which would increase the combined expression of BMAL1 heterodimer partner even further. Of note, the rhythm of *Clock* expression in the wild-type (WT) liver was of lower amplitude than that of *Arntl* and *Npas2* (Figure 4B), and as a result, the constitutive overexpression induced by REV-ERB DKO was less severe. This lower dynamic range of expression may explain why we did not observe an upregulation of *Clock* in our REV-ERB DKO SCN slices. Taken together, our results show that REV-ERB nuclear receptors have a critical, tissue-autonomous role in maintaining circadian period length in multiple tissues, due to their ability to repress the combined levels of BMAL heterodimer partners NPAS2 and CLOCK. This ability of REV-ERBs to tune the endogenous clock period length to near 24 h prevents discordance between internal and external rhythms. At the same time, tissue-specific clock properties, such as phase coherence, are modulated by REV-ERBs in a tissue-specific manner.

### Limitations of the study

Our study has several technical limitations. The low signal strength of bioluminescent fusion reporters like PER2::LUC presents challenges when attempting to analyze microscopy data at the cellular level. While we have focused our analyses on roughly cell-sized pixels, these pixels do not correspond in a 1:1 fashion with individual cells. Biological tissues such as the SCN and the liver maintain complex cellular architecture *in vivo* that is only partially preserved in slice culture, which may impact the coherence of circadian signals when cultured. While our slice culture system allows us to assess *ex vivo* the circadian properties of these tissues free from external influences, those influences are numerous *in vivo*, and thus it remains difficult to fully predict how what we observe *ex vivo* impacts the circadian network *in vivo*. In the case of SCN, the cellular identity of the cultured slice is expected to remain largely consistent throughout culture, but the liver slices may undergo dedifferentiation over time in culture. Finally, the small size of the cultured SCN and liver limit the amount of usable RNA that can be extracted from the samples, preventing wider-scale analysis of gene expression.

## RESOURCE AVAILABILITY

### Lead contact

Further information and requests for resources and reagents should be directed to the lead contact, Mitchell A. Lazar (lazar@pennmedicine.upenn.edu).

### Materials availability

Reagents generated in this study will be made available upon request, but we may require a payment and/or completed materials transfer agreement if there is a potential for commercial application.

### Data and code availability

- The data (luminescence traces, microscope timelapses, qPCR results, and RNA-seq quantifications) have been deposited at Zenodo and are publicly available at https://doi.org/10.5281/zenodo.17127324 as of the date of publication.
- All original code has been deposited at Zenodo and is publicly available at https://doi.org/10.5281/zenodo.17127324 as of the date of publication.
- Any additional information required to reanalyze the data reported in this paper is available from the lead contact upon request.

## STAR★METHODS

### EXPERIMENTAL MODEL AND STUDY PARTICIPANT DETAILS

#### Animals

All animal work reported in this study was approved by the University of Pennsylvania Perelman School of Medicine Institutional Animal Care and Use Committee, in accordance with NIH guidelines. Mice were bred and maintained on a 12:12 h light:dark cycle with *ad libitum* access to food and water. All mice used (REV-ERBα and β floxed mice with and without heterozygous expression of the fusion protein reporter PER2::LUC) were maintained on a C57Bl/6J background. Male and female mice were used (see figure captions for specific usage per experiment). We did not observe any association between sex and SCN period length (Figure S1). In all other measurements, low within-sex sample sizes limited our ability to statistically test associations between sex and experimental outputs. All mice used were between 8 and 22 weeks old at the time of sacrifice.

## METHOD DETAILS

### SCN organotypic slice preparation

Male and female mice were sacrificed by cervical dislocation followed by rapid removal of the brain. Brain tissue was incubated in cold HBSS-based slicing solution (per 1 L solution: 100 mL 10× Ca/Mg-free HBSS, 10 mL 10,000 U/mL Penicillin-Streptomycin, 5 mL 7.5% sodium bicarbonate, 2.38 g HEPES) during slicing with a Campden Instruments 7000smz-2 vibratome. Slices were made at 200 μm followed by immediate microdissection of the SCN. SCN tissue was transferred to a nitrocellulose membrane (Millipore PICM0RG50) inside a 35 mm dish with 1.2 mL of luciferin-containing (except when used for calcium imaging, see below), DMEM-based media with B-27 supplement (per 1 L solution: 12.78 g high-glucose (4.5 g/L), phenol-red-free, L-glutamine free DMEM powder, 0.5 g D-glucose, 4.7 mL 7.5% sodium bicarbonate, 2.38 g HEPES, 2.5 mL Penicillin-Streptomycin, 10 mL GlutaMAX). After transferring to the membrane, SCN slices received 1.5–2 μL of the indicated virus or virus mixture (see below). Culture dishes were then sealed with Microseal ‘B’ sealing tape (Bio-Rad) and incubated at 37°C.

### Tail vein injection

Male and female mice were restrained and tails warmed by water bath prior to injection of 200 μL of the specified virus (see below) into the tail vein. Mice were given at least 13 days for viral transduction in the liver.

### Liver organotypic slice preparation

Male and female mice were sacrificed by cervical dislocation followed by rapid removal of one lobe of the liver. Liver tissue was incubated and sliced using the same procedure as the SCN (see above). Slices were made at 200 μm followed by 3-mm circular punch biopsy (Thomas Scientific) to make consistently-sized tissue cultures. Liver slice punches were cultured using the same procedures as the SCN (see above).

### Luminometry

SCN and liver slices expressing PER2::LUC were recorded in a LumiCycle 32 luminometer (Actimetrics) housed within a 37°C incubator without CO_2_, with readings every 10 min.

### Microscopy

SCN and liver slices expressing PER2::LUC were recorded using a Zaber MVR digital microscope with a 37°C stage-top incubator using a 10× objective and a Hamamatsu ImagEM C9100–13 CCD camera in normal CCD mode. For PER2::LUC recordings, SCN and liver slices were imaged using μManager software (SCN: 4 × 4 binning, 9 min exposure, every 1 h; liver: 4 × 4 binning, 14 min exposure, every 1 h). SCN slices for calcium imaging were made from mice not expressing PER2::LUC (and using media not containing luciferin), and were imaged using a GFP filter-set and blue light excitation with 125 ms exposure, 1 × 1 binning every 20 min.

### Viral transduction: SCN

#### Knockout induction

SCN REV-ERB DKO was induced in REV-ERBα and β floxed mice using AAV(PHP.eB)-Syn-Cre (SignaGen, SL116012, “Syn-Cre”), while Control SCN slices received AAV(PHP.B)-CMV-Null (SignaGen, SL101490, “CMV-Null”). Slices receiving these viruses alone were given 72 h to transduce.

#### Calcium imaging

SCN slices used for calcium imaging received either CMV-Null or Syn-Cre along with AAV(PHP.eB)-Syn-jGCaMP7s-WPRE (Addgene 104487) at a 1:1 ratio. Slices receiving these viruses were given 10 days to transduce the GCaMP expression stably.

#### shRNA experiments

SCN slices used for shRNA experiments received either CMV-Null or Syn-Cre along with either AAV(PHPe.B)-U6-shCtrl (VectorBuilder, “shCtrl”, target sequence: CCTAAGGTTAAGTCGCCCTCG), a 1:1:1 mixture of AAV(PHP.eB)-U6-shClock[#1–3] (VectorBuilder, “shClock”, target sequences: CGGATGATAGAGGCAAATATT [#1], GAGAACATTCAGAGGTTTATA [#2], GTGCTTCAGATGTCCATTAAA [#3]), a 1:1 mixture of AAV(PHP.eB)-U6-shNpas2[#1–2] (VectorBuilder, “shNpas2”, target sequences: GCAAGAACATTCCGAAGTTTA [#1], CCAGCAGCCATCAGGGTAATA [#2]), or a combination of the shClock and shNpas2 mixtures. For shCtrl treatments, the ratio was 40% CMV-Null or Syn-Cre and 60% shCtrl. For shClock treatment, the ratio was 40% CMV-Null or Syn-Cre, 36% shClock[#1–3], and 24% slicing solution. For shNpas2 treatment, the ratio was 40% CMV-Null or Syn-Cre, 24% shNpas2[#1–2], and 36% slicing solution. For the combination shNpas2 and shClock treatment, the ratio was 40% CMV-Null or Syn-Cre, 24% shNpas2 [#1–2], and 36% shClock[#1–3]. Slices receiving these virus combinations were given 5 days to transduce. shRNA viruses were made through laboratory harvest of AAV from plasmid constructs designed and cloned using VectorBuilder. shArntl experiments used a ratio of 40% CMV-Null or Syn-Cre, 60% AAV(PHP.eB)-U6-shArntl[#1–3] (VectorBuilder, “shArntl”, target sequences: TCTTCAAGATCCTCAATTATA [#1], GCAGTATCAAAGTGCATTAAT [#2], GACGAACTGAAACACCTAATT [#3].

#### Overexpression experiments

Overexpression experiments were conducted by subcloning *Npas2* (Origene MR221661), *Clock* MR226315, or *Arntl* (Addgene #31367) into an AAV-CAG backbone (Addgene, Plasmid #100844) followed by packaging into AAV PHP.eB virus (lab made). CAG-GFP (Addgene #37825) or CMV-Null was used as a control. Viruses were applied to slices as 1.75–2 μL drops at the time of slicing.

### Viral transduction: Liver

Liver REV-ERB DKO was induced in REV-ERBα and β floxed mice using tail vein-injected AAV(8)-TBG-Cre, while Control mice received tail vein injection of AAV(8)-TBG-Gfp. Following injection, mice were used for organotypic slice preparation on the 14^th^ day following injection, at earliest.

### Pharmacological treatments

#### PF670462 treatments

Organotypic SCN slices were prepared as described above and recorded for 7 days. On the 8^th^ day (hour 179.89), slices were removed from the LumiCycle one at a time, seals removed, and 1.2 μL of 1 mM PF670462 dissolved in DMSO (final concentration: 1 μM) or 1.2 μL DMSO vehicle was added to each dish below the membrane before replacing in the LumiCycle for further recording.

#### Cycloheximide treatments

Organotypic SCN slices were prepared as described above and recorded for 5 days. On the 6^th^ day, slices were removed from the LumiCycle close to the peak expression time of PER2::LUC, seals removed, and 1.2 μL of 10 mg/mL cycloheximide dissolved in DMSO was added to the media below the membrane (final concentration: 10 μg/mL). Slices were replaced in the LumiCycle for further recording.

### Gene expression

SCN slices were collected near the peak of PER2::LUC expression (CT 12) directly into lysis buffer and RNA extracted using an RNeasy Plus Micro Kit (Qiagen). cDNA was synthesized using (Applied Biosystems). qPCR was performed using SYBR Green universal master mix (Applied Biosystems) in a QuantStudio 6 (Applied Biosystems) device. Quantification was performed using the ΔΔCt method, with ΔCt against the housekeeping gene *Rpl19* (F: ATGAGTATGCTCAGGCTACAGA, R: GCATTGGCGATTTCATTGGTC) and ΔΔCt against the Control SCN slice mean for each gene. *Nr1d1* (F: GTCTCTCCGTTGGCATGTCT, R: CCAAGTTCATGGCGCTCT) and *Nr1d2* (F: TTCTACTGTGTAAAGTCTGTGGG, R: CTGGATGTTTTGCTGAATGCTC) primers targeted the floxed exons excised upon Cre-mediated recombination in the REV-ERBα and β floxed line. Primers for remaining genes: *Arntl* (F: TAGGATGTGACCGAGGGAAG, R: TCAAACAAGCTCTGGCCAAT), *Npas2* (F: ATGTTCGAGTGGAAAGGAGAC, R: CAAGTGCATTAAAGGGCTGTG), and *Clock* (F: TCTCAAGGAAGCACTGGAAAG, R: CAGTAGGGATCTTTGTCGGTG). RNA-seq data from Guan et al.^14^ (GSE143524) were processed with Salmon 1.9.0 using mm10. Salmon quants were imported to R using tximport, then processed using edgeR and limma. Sinusoidal fits were generated using linear-form sine wave + cosine wave linear models.

#### Schematics

Schematics were created using BioRender.

## QUANTIFICATION AND STATISTICAL ANALYSES

### Whole-slice PER2::LUC luminescence and calcium fluorescence analysis

#### Trace arrangement

For luminometer recordings, raw luminescence traces from the were exported to R. For calcium imaging microscopy, TIFF files were loaded into R as arrays^35^ and the mean fluorescence intensity for each frame was used to generate a longitudinal trace.

#### Detrending

Aberrant single counts were filtered by excluding readings that deviated from a 2-h rolling mean smoothed trace by more than 8 times the mean deviation for that trace. Detrended traces were produced by subtracting a 24-h rolling mean from the raw trace.

#### Period measurement

Period was measured over the indicated time window (see figure captions) of the detrended traces using the Lomb-Scargle Periodogram as implemented in the Spectr R package.^36^

#### Amplitude measurement

Amplitude was measured as the difference between peak and trough values for each cycle, divided by the root-mean-square error of the trace. Peak and trough times were assigned by identifying sign changes in the slope of a sliding, 8-h linear fit of the smoothed (2-h rolling mean) trace. Peak and trough values were assigned by taking the value of the smoothed trace at these inflection times. Root-mean-square error for each trace was calculated as the square root of the mean squared difference between the detrended trace and the smoothed trace.

#### Half-life estimation

Half-life was estimated by normalizing 12 h of luminescence trace 2 h after cycloheximide (CHX) application from 0 to 1. Each normalized trace was then fit with an exponential decay model (*y* ~1 *e^(−*t*/*k*)^, where y is the normalized luminescence signal, *t* is the time in hours, and *k* is the mean lifetime (seeding value *k* = 3). Half-life was calculated as the mean lifetime *k* multiplied by log(2).

#### PER2::LUC bibned-pipel mimroscopy analysis

Prior to loading into R, SCN recordings were opened and trimmed to 190 frames. CCD noise was eliminated using a 2-frame minimization, followed by a whole-recording mean luminescence projection. From this projection, the SCN itself was manually outlined and a binary mask was created. The original raw recording was then multiplied by this binary mask and saved for export into R. For liver recordings, raw recordings were loaded directly into R. TIFF files were loaded into R as arrays and pixels were further binned by a factor of 4. Peaks and troughs of the overall recording were identified using the sliding slope method described above, and then the peak times of each binned pixel were identified using the findpeaks function of the pracma^37^ R package. Pixel peak times were assigned to a cycle number based on the overall luminescence trace peak times, and the peak time of each individual binned pixel was quantified relative to the mean peak time for that cycle. These relative peak times were used to generate histograms and to calculate phase distribution using the median absolute deviation (MAD) for each cycle. ***p < 0.001, **0.001 < *p* < 0.01, *0.01 < *p* < 0.05, ^ns^*p* > 0.05.

## Supplementary Material

1

SUPPLEMENTAL INFORMATION

Supplemental information can be found online at https://doi.org/10.1016/j.celrep.2025.116437.

## Figures and Tables

**KEY RESOURCES TABLE:** 

REAGENT or RESOURCE	SOURCE	IDENTIFIER

Bacterial and virus strains		

AAV(PHP.eB)-Syn-Cre	SignaGen	SL116012
AAV(PHP.B)-CMV-Null	SignaGen	SL101490
AAV(PHP.eB)-Syn-jGCaMP7s-WPRE	Addgene	104487
AAV8-TBG-Gfp	Penn Vector Core	N/A
AAV8-TBG-Cre	Penn Vector Core	N/A
AAV(PHP.eB)-CAG-Npas2	This paper	N/A
AAV(PHP.eB)-CAG-Arntl	This paper	N/A
AAV(PHP.eB)-CAG-Clock	This paper	N/A
AAV(PHP.eB)-U6-shRNA (Npas2, Clock, Arntl)	This paper	N/A

Chemicals, peptides, and recombinant proteins		

HBSS	Thermo Scientific	14185052
Penicillin-Streptomycin	Thermo Scientific	15140122
B-27 Plus	Thermo Scientific	A3582801
GlutaMAX	Thermo Scientific	35050061
Cycloheximide	Cell Signaling Technologies	2112S
PF670462	Sigma	SML0795-5MG
DMEM	Corning	90-013-PB

Deposited data		

Control and Hepatocyte-specific REV-ERB DKO liver (RNA-seq)	Guan et al.^14^	GEO: GSE143524

Experimental models: Organisms/strains		

Nr1d1^fl/fl^Nr1d2^fl/fl^	Guan et al.^14^	N/A
Nr1d1^fl/fl^Nr1d2^fl/fl^Per2^per2::Luciferase/+^	Adlanmerini et al.^12^	N/A

Oligonucleotides		

Rpl19-F: ATGAGTATGCTCAGGCTACAGA	IDT	N/A
Rpl19-R: GCATTGGCGATTTCATTGGTC	IDT	N/A
Nr1d1-F: GTCTCTCCGTTGGCATGTCT	IDT	N/A
Nr1d1-R: CCAAGTTCATGGCGCTCT	IDT	N/A
Nr1d2-F: TTCTACTGTGTAAAGTCTGTGGG	IDT	N/A
Nr1d2-R: CTGGATGTTTTGCTGAATGCTC	IDT	N/A
Arntl-F: TAGGATGTGACCGAGGGAAG	IDT	N/A
Arntl-R: TCAAACAAGCTCTGGCCAAT	IDT	N/A
Npas2-F: ATGTTCGAGTGGAAAGGAGAC	IDT	N/A
Npas2-R: CAAGTGCATTAAAGGGCTGTG	IDT	N/A
Clock-F: TCTCAAGGAAGCACTGGAAAG	IDT	N/A
Clock-R: CAGTAGGGATCTTTGTCGGTG	IDT	N/A

Recombinant DNA		

pAAV-U6-shCtrl (target sequence: CCTAAGGTTAAGTCGCCCTCG)	VectorBuilder	N/A
pAAV-U6-shClock[#1–3] (target sequences: CGGATGATAGAGGCAAATATT [#1], GAGAACATTCAGAGGTTTATA [#2], GTGCTTCAGATGTCCATTAAA [#3])	VectorBuilder	N/A
AAV(PHP.eB)-U6-shNpas2[#1–2] (target sequences: GCAAGAACATTCCGAAGTTTA [#1], CCAGCAGCCATCAGGGTAATA [#2])	VectorBuilder	N/A
AAV(PHP.eB)-U6-shArntl[#1–3] (target sequences: TCTTCAAGATCCTCAATTATA [#1], GCAGTATCAAAGTGCATTAAT [#2], GACGAACTGAAACAC CTAATT [#3])	VectorBuilder	N/A
Npas2 (BC109166) Mouse Tagged ORF Clone	OriGene	MR221661
Clock (NM_007715) Mouse Tagged ORF Clone	OriGene	MR226315
pBMPC3	Addgene	31367
pAAV.CAG.GCaMP6s.WPRE.SV40	Addgene	100844
pAAV-CAG-GFP	Addgene	37825

Software and algorithms		

μManager	http://micro-manager.org	RRID:SCR_000415
Salmon v.1.9.0	Patro et al.^34^	RRID:SCR_017036
Tximport	https://github.com/mikelove/tximport	RRID:SCR_016752
edgeR	http://bioconductor.org/packages/edgeR/	RRID:SCR_012802
Limma	http://bioinf.wehi.edu.au/limma/	RRID:SCR_010943
BioRender	http://biorender.com	RRID:SCR_018361
Ijtiff	Nolan et al.^35^	N/A
Spectr	Tackenberg & Hughey^36^	N/A
Original code	This manuscript	10.5281/zenodo.17127324

Other		

Cell culture membranes	Millipore Sigma	PICM0RG50
Microseal ‘B’ sealing tape	Bio-Rad	MSB1001B
3.0 mm biopsy punch	Thomas Scientific	1190R25
LumiCycle 32 luminometer	Actimetrics	N/A
Vibratome	Campden Instruments	7000smz-2
Digital microscope	Zaber	MVR
CCD camera	Hamamatsu	C9100-13

## Highlights

- REV-ERB nuclear receptors maintain normal circadian period in brain and liver
- Period shortening due to REV-ERB deletion is not through destabilization of PER2
- *Npas2* is markedly derepressed by loss of REV-ERBs
- Depletion of *Npas2* and *Clock* rescues the short period phenotype
