# Supplementary material for: Normal circadian period length requires repression of Npas2 by REV-ERB nuclear receptors
Source: Cell Rep. Author manuscript; Available in PMC 2025 Nov 12. (PMC12608050; doi:10.1016/j.celrep.2025.116437)
Supplement: 1 [file NIHMS2120121-supplement-1.pdf]

Cell Reports, Volume *44*

Supplemental information

Normal circadian period length requires repression of *Npas2* by REV-ERB nuclear receptors

Michael C. Tackenberg, Kristina M. Heliodoro, Lily C. Melink, Yifan Liu, Kun Zhu, and Mitchell A. Lazar

SUPPLEMENTAL FIGURES

Figure S1

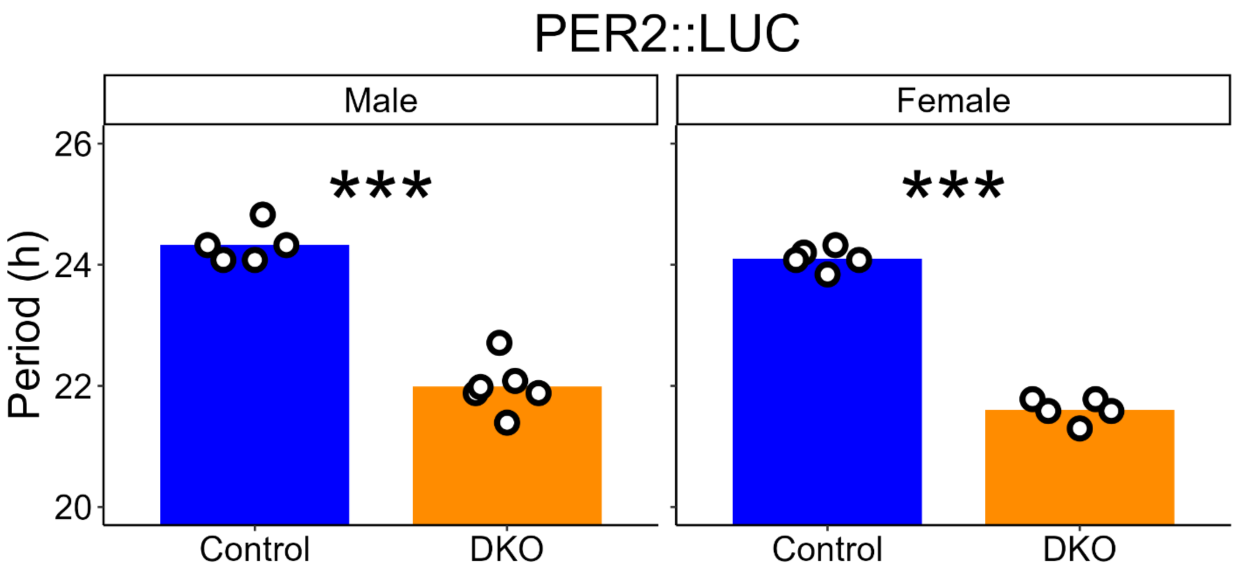

Figure S1. REV-ERB DKO period shortening in the SCN is not sex-specific. Related to Figure 1.

Period length of the PER2::LUC rhythm for male (left) and female (right) control (blue, male mean ± SEM: 24.33 ± 0.14 h, n = 5, female mean ± SEM: 24.10 ± 0.08 h, n = 5) and REV-ERB DKO (orange, male mean ± SEM: 21.99 ± 0.17 h, n = 6, female mean ± SEM: 21.61 ± 0.09 h, n = 5) SCN slices shown in **Fig. 1C** as measured by Lomb-Scargle Periodogram on hours 72 through 168 of the recording (two-way ANOVA, p(main effect of sex) = 0.0364, p(REV-ERB Genotype) < 0.0001, p(interaction) = 0.5561; Šidák's multiple comparisons test: p(male, Control – DKO) < 0.0001, p(female, Control – DKO) < 0.0001, p(Control, male – female) = 0.2634, p(DKO, male – female) = 0.0533). Bar height represents mean values.

Figure S2

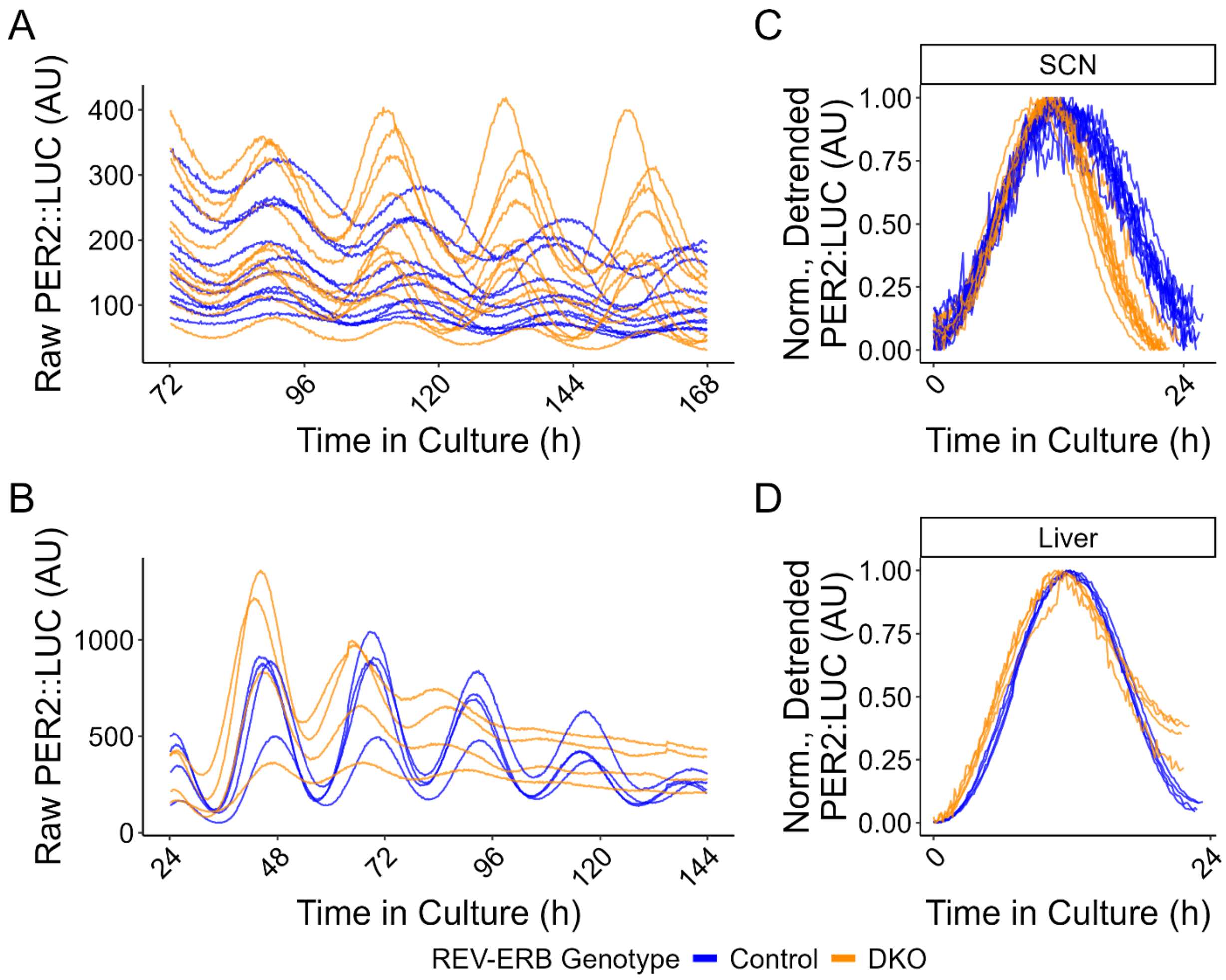
A
Raw PER2::LUC (AU)
400
300
200
100
72
96
120
144
168
Time in Culture (h)
B
Raw PER2::LUC (AU)
1000
500
0
24
48
72
96
120
144
Time in Culture (h)
C
SCN
Norm., Detrended PER2:LUC (AU)
1.00
0.75
0.50
0.25
0.00
0
24
Time in Culture (h)
D
Liver
Norm., Detrended PER2:LUC (AU)
1.00
0.75
0.50
0.25
0.00
0
24
Time in Culture (h)
REV-ERB Genotype
Control
DKO

Figure S2. PER2::LUC signal remains elevated in Control and REV-ERB DKO SCN and liver slices. Related to Figure 3.

(A-B) Raw PER2::LUC signal for each control (blue) and REV-ERB DKO (orange) SCN (A) and liver (B) slice.

(C-D) The final cycle of PER2::LUC for the SCN (C) and liver (D), normalized from 0 to 1.

Figure S3

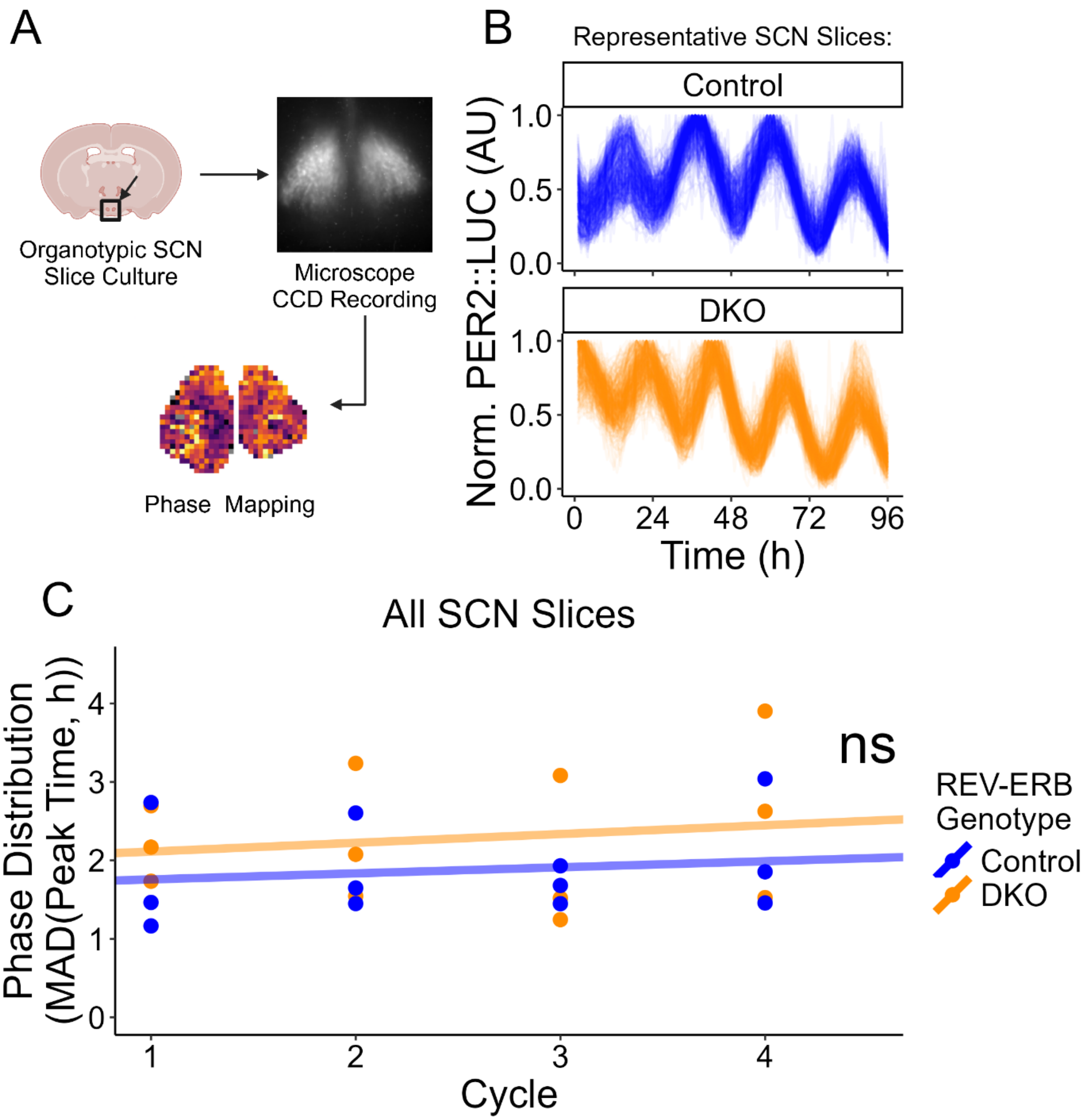
A
Organotypic SCN
Slice Culture
Microscope
CCD Recording
Phase Mapping
B
Representative SCN Slices:
Control
DKO
Norm. PER2::LUC (AU)
1.0
0.5
0.0
0
24
48
72
96
Time (h)
C
All SCN Slices
Phase Distribution
(MAD(Peak Time, h))
0
1
2
3
4
ns
1
2
3
4
Cycle
REV-ERB
Genotype
Control
DKO

Figure S3. REV-ERB DKO does not impact phase coherence in the SCN. Related to Figure 3.

(A) Schematic view of the SCN real-time bioluminescence imaging of PER2::LUC signal by CCD microscope. Recording started on the 4th day after slicing.

(B) Individual PER2::LUC traces from pixels from a representative control (top, blue) and REV-ERB DKO (bottom, orange) SCN slice.

(C) Phase distribution as measured by median absolute deviation (MAD) of relative peak time values for control (blue) and REV-ERB DKO (orange) SCN slices (extra sum of squares F test for unshared slope, $p = 0.9009$). Line represents linear fit of the data.

Figure S4

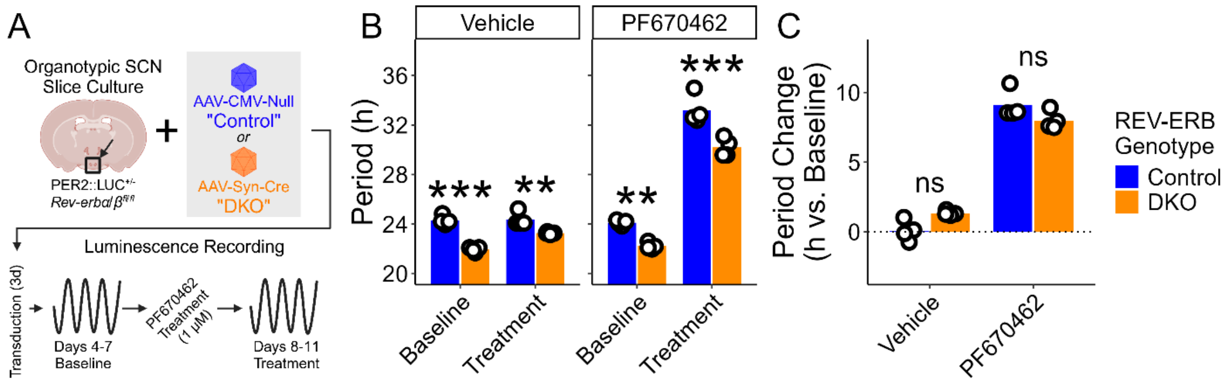

A
Organotypic SCN Slice Culture
PER2::LUC+/-
Rev-erbα/β fl/fl
AAV-CMV-Null
"Control"
or
AAV-Syn-Cre
"DKO"
Luminescence Recording
Transduction (3d)
Days 4-7
Baseline
PF670462 Treatment (1 µM)
Days 8-11
Treatment
B
Vehicle
PF670462
Period (h)
36
32
28
24
20
***
**
**
***
Baseline
Treatment
Baseline
Treatment
C
Period Change (h vs. Baseline)
10
5
0
ns
ns
Vehicle
PF670462
REV-ERB Genotype
Control
DKO

Figure S4. Pharmacological inhibition of CK1δ/ε by PF670462 does not restore REV-ERB DKO period length to control levels in SCN slices. Related to Figure 4.

(A) Schematic view of the SCN organotypic slice preparation and CK1δ/ε inhibitor (PF670462) application.

(B) Period length of PER2::LUC rhythms as measured by Lomb-Scargle Periodogram from control (blue) and REV-ERB DKO (orange) SCN slices before ("Baseline", hours 72 through 168) and after ("Treatment", hours 189 through 285) treatment with either DMSO vehicle or 1 μM PF670462. Vehicle-treated groups: Baseline mean ± SEM: Control (n = 4, 2 male, 2 female) 24.30 ± 0.19 h, DKO (n = 4, 2 male, 2 female) 21.93 ± 0.10; Treatment mean ± SEM: Control 24.36 ± 0.29 h, DKO 23.26 ± 0.05 h. Two-way ANOVA, *p*(treatment window) = 0.0020, *p*(REV-ERB genotype) < 0.0001, *p*(interaction) = 0.0041. Šidák's multiple comparisons test: *p*(baseline, Control – DKO) < 0.0001, *p*(treatment, Control – DKO) = 0.0018. PF670462-treated groups: Baseline mean ± SEM: Control (n = 4, 2 male, 2 female) 24.11 ± 0.10 h, DKO (n = 4, 2 male, 2 female) 22.21 ± 0.14 h; Treatment mean ± SEM: Control 33.19 ± 0.60 h, DKO 30.20 ± 0.38 h. Two-way ANOVA *p*(treatment window) < 0.0001, *p*(REV-ERB genotype) < 0.0001, *p*(interaction) = 0.1595. Šidák's multiple comparisons test: *p*(baseline, Control – DKO) = 0.0063, *p*(treatment, Control – DKO) = 0.0002. Bar heights represent mean values.

(C) Change in period length for each slice after the indicated treatment relative to the baseline period. Vehicle mean ± SEM: Control 0.07 ± 0.37 h, DKO 1.33 ± 0.09 h, PF670462 mean ± SEM: Control 9.08 ± 0.52 h, DKO 7.97 ± 0.33 h. Two-way ANOVA: *p*(REV-ERB genotype) = 0.8216, *p*(treatment) < 0.0001, *p*(interaction) = 0.0068. Šidák's multiple comparisons test: *p*(Vehicle, Control – DKO) = 0.0584, *p*(PF670462, Control – DKO) = 0.1041. Bar heights represent mean values.

Figure S5

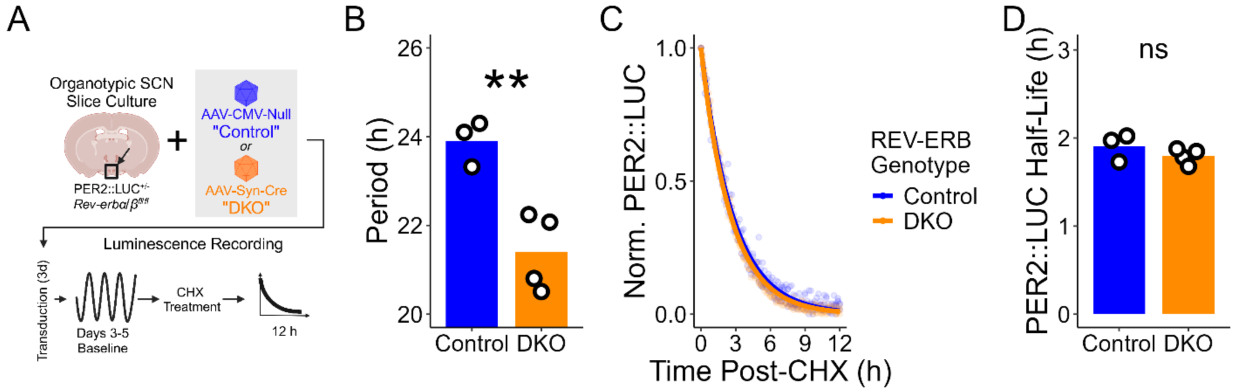

A
Organotypic SCN Slice Culture
+
AAV-CMV-Null
"Control"
or
AAV-Syn-Cre
"DKO"
PER2::LUC+/-
Rev-erbα/β fl/fl
Luminescence Recording
Transduction (3d)
Days 3-5 Baseline
CHX Treatment
12 h
B
**
Period (h)
26
24
22
20
Control
DKO
C
Norm. PER2::LUC
1.0
0.5
0.0
0
3
6
9
12
Time Post-CHX (h)
REV-ERB Genotype
Control
DKO
D
ns
PER2::LUC Half-Life (h)
3
2
1
0
Control
DKO

Figure S5. REV-ERB DKO has no effect on SCN PER2 half-life. Related to Figure 4.

(A) Schematic view of the cycloheximide (CHX) application experiment.

(B) Period length of control (blue, mean ± SEM: 23.91 ± 0.30 h, n = 3, 2 male, 1 female) and REV-ERB DKO (orange, mean ± SEM: 21.41 ± 0.44 h, n = 4, 2 male, 2 female) prior to treatment as measured by Lomb-Scargle Periodogram on hours 72 to 130 of the recording (t-test, $p = 0.0075$). Bar heights represent mean values.

(C) Normalized luminescence decay of control (blue) and REV-ERB DKO (orange) SCN slices following CHX treatment.

(D) Estimated PER2::LUC half-life as calculated by exponential decay fit of Control (blue, mean ± SEM: 1.91 ± 0.09 h) and REV-ERB DKO (orange, mean ± SEM: 1.80 ± 0.04 h). SCN slices (t-test, $p = 0.2791$). Bar heights represent mean values.

Figure S6

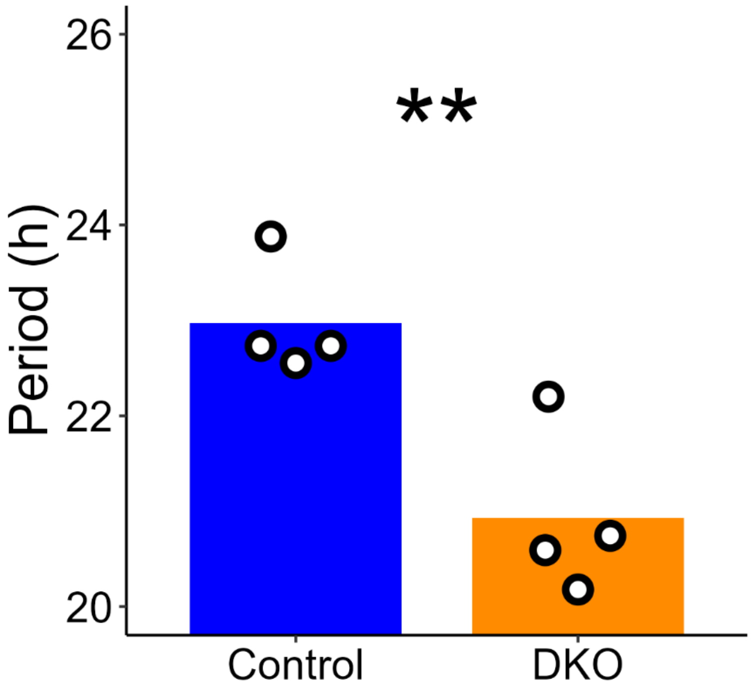

Figure S6. REV-ERB DKO period is shortened in the SCN prior to RNA extraction. Related to Figure 4.

Period length of control (blue, mean ± SEM: 22.97 ± 0.30 h, n = 4; 2 male, 2 female) and REV-ERB DKO (orange, mean ± SEM: 20.93 ± 0.44 h, n = 4, 1 male, 3 female) SCN slices prior to collection, as measured by Lomb-Scargle Periodogram on hours 72 through 129 of the recording (t-test, $p = 0.0087$).

Figure S7

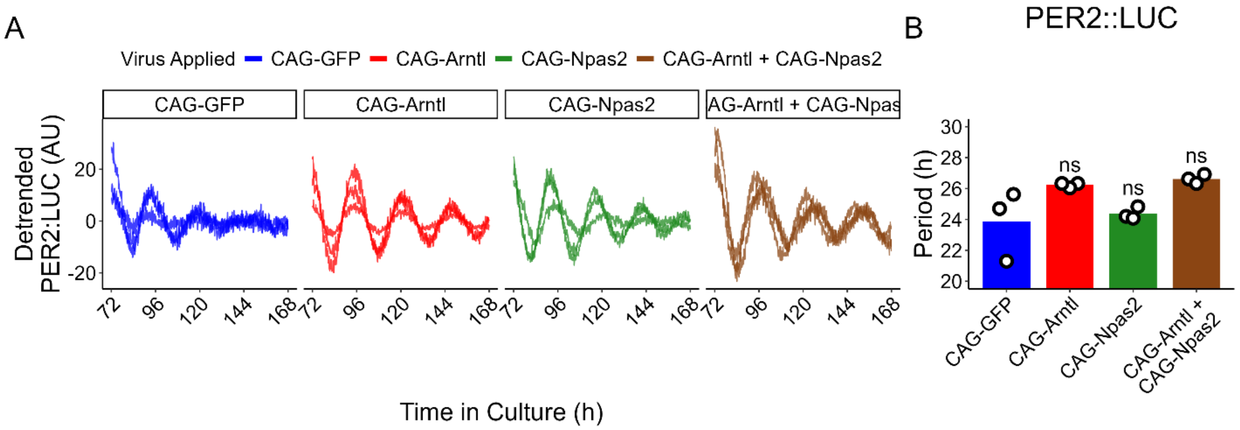
A
Virus Applied
CAG-GFP
CAG-Arntl
CAG-Npas2
CAG-Arntl + CAG-Npas2
CAG-GFP
CAG-Arntl
CAG-Npas2
AG-Arntl + CAG-Npas
Detrended PER2:LUC (AU)
20
0
-20
72
96
120
144
168
Time in Culture (h)
B
PER2::LUC
Period (h)
30
28
26
24
22
20
ns
ns
ns
CAG-GFP
CAG-Arntl
CAG-Npas2
CAG-Arntl + CAG-Npas2

Figure S7. Overexpression of *Bmal1* does not mimic the REV-ERB DKO period shortening in SCN slices. Related to Figure 6.

(A) Individual detrended PER2::LUC traces of CAG-GFP- (blue), CAG-Arntl- (red), CAG-Npas2- (green), and CAG-Arntl + CAG-Npas2-treated (brown) SCN slices.

(B) Period length of the PER2::LUC rhythm for CAG-GFP- (blue, mean ± SEM: 23.87 ± 1.32 h, n = 3, 2 male, 1 female), CAG-Arntl- (red, mean ± SEM: 26.23 ± 0.10 h, n = 3, 2 male, 1 female), CAG-Npas2- (green, mean ± SEM: 24.37 ± 0.23 h, n = 3, 2 male, 1 female), and CAG-Arntl + CAG-Npas2-treated (brown, mean ± SEM: 26.62 ± 0.17 h, n = 3, 1 male, 2 female) SCN slices as measured by Lomb-Scargle Periodogram. Each experimental group was compared to the CAG-GFP-treated group using Dunnett's Test with Šidák's correction (*p*(CAG-Arntl) = 0.2507, *p*(CAG-Npas2) = 0.9994, *p*(CAG-Arntl + Npas2) = 0.1427. Bar height represents mean value.

Figure S8

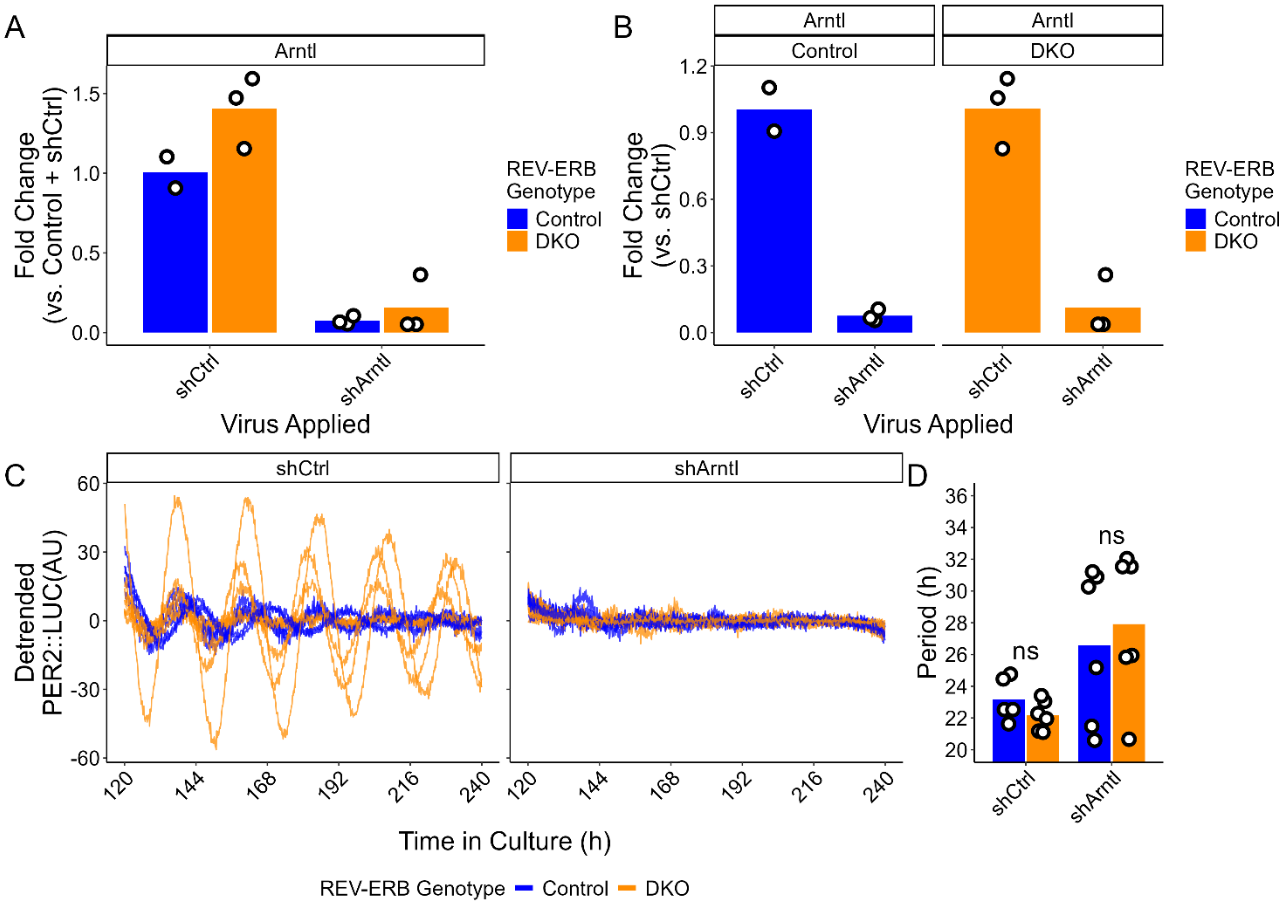

A
Arntl
Fold Change (vs. Control + shCtrl)
1.5
1.0
0.5
0.0
shCtrl
shArntl
Virus Applied
REV-ERB Genotype
Control
DKO
B
Arntl
Control
Arntl
DKO
Fold Change (vs. shCtrl)
1.2
0.9
0.6
0.3
0.0
shCtrl
shArntl
shCtrl
shArntl
Virus Applied
REV-ERB Genotype
Control
DKO
C
shCtrl
shArntl
Detrended PER2::LUC(AU)
60
30
0
-30
-60
120
144
168
192
216
240
Time in Culture (h)
REV-ERB Genotype
Control
DKO
D
Period (h)
36
34
32
30
28
26
24
22
20
ns
ns
shCtrl
shArntl

Figure S8. Knockdown of *Bmal1* does not rescue the REV-ERB DKO period shortening in SCN slices. Related to Figure 5.

(A) *Arntl* expression for shCtrl- and shArntl-treated Control (blue) and REV-ERB DKO (orange) SCN slices relative to Control + shCtrl.

(B) *Arntl* expression for shCtrl- and shArntl-treated Control (blue) and REV-ERB DKO (orange) SCN slices relative to the shCtrl-treated mean for each group.

(C) Individual detrended PER2::LUC traces of control (blue) and REV-ERB DKO (orange) SCN slices treated with shCtrl (left) or shArntl (right).

(D) Period length of the PER2::LUC rhythm for Control (blue) and REV-ERB DKO (orange) SCN slices treated with shCtrl (Control mean ± SEM: 23.18 ± 0.61 h, n = 5, 3 male, 2 female; DKO mean ± SEM: 22.16 ± 0.39 h, n = 6, 4 male, 2 female) or shArntl (Control mean ± SEM: 26.60 ± 1.98 h, n = 6, 2 male, 4 female; DKO mean ±SEM: 27.92 ± 1.86 h, n = 6, 2 male, 4 female) as measured by Lomb-Scargle Periodogram (two-way ANOVA, *p*(REV-ERB Genotype) = 0.9192, *p*(shRNA treatment) = 0.0054. Šidák's multiple comparisons test: *p*(shCtrl, Control – DKO) = 0.8672, *p*(shArntl, Control – DKO) = 0.7704. Bar height represents mean value. Gene expression calculated from RNA extracted from each slice from one of two trials included in panels C and D.

Figure S9

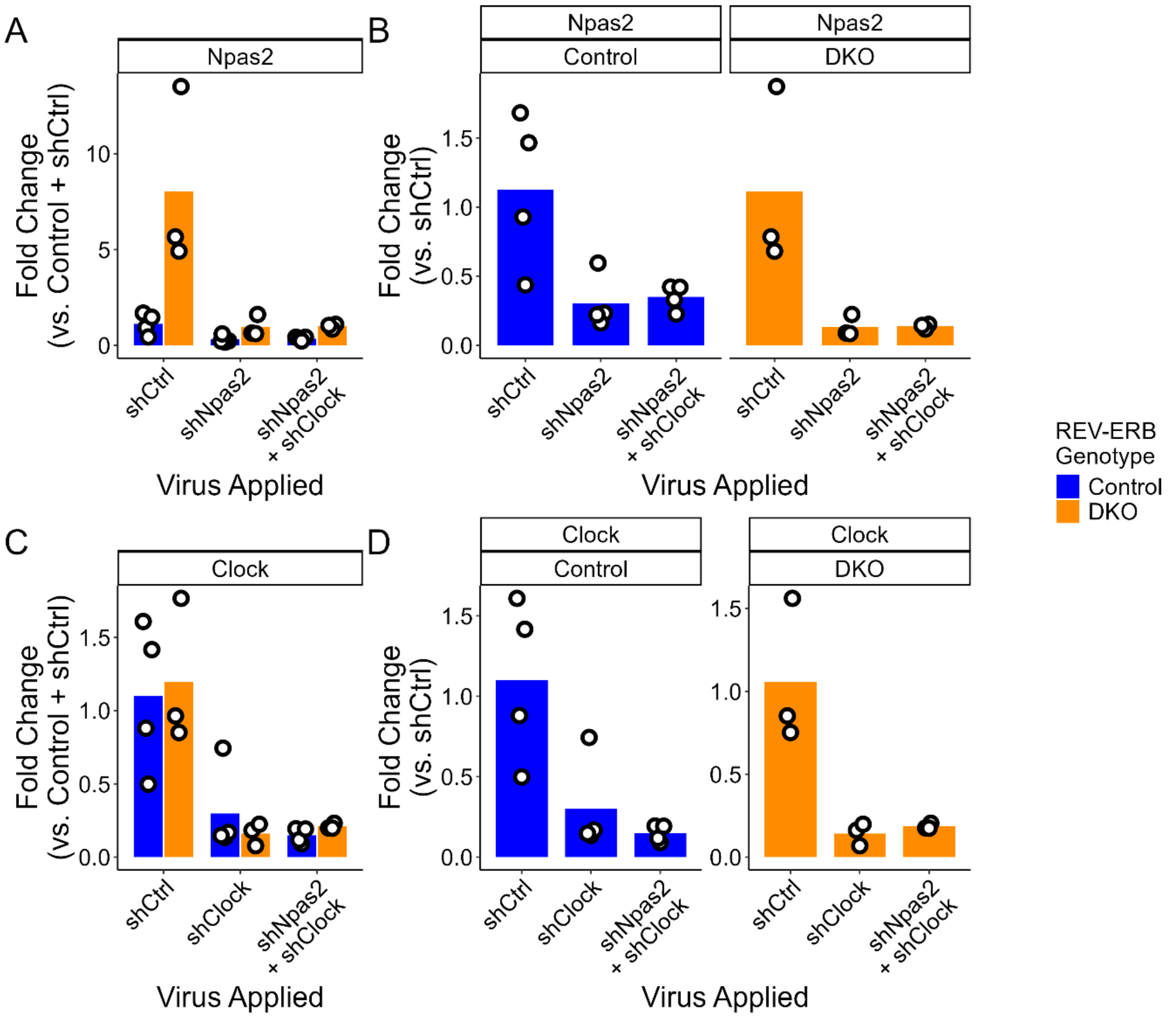
A
Npas2
Fold Change
(vs. Control + shCtrl)
0
5
10
shCtrl
shNpas2
shNpas2
+ shClock
Virus Applied
B
Npas2
Control
Npas2
DKO
Fold Change
(vs. shCtrl)
0.0
0.5
1.0
1.5
shCtrl
shNpas2
shNpas2
+ shClock
Virus Applied
REV-ERB
Genotype
Control
DKO
C
Clock
Fold Change
(vs. Control + shCtrl)
0.0
0.5
1.0
1.5
shCtrl
shClock
shNpas2
+ shClock
Virus Applied
D
Clock
Control
Clock
DKO
Fold Change
(vs. shCtrl)
0.0
0.5
1.0
1.5
shCtrl
shClock
shNpas2
+ shClock
Virus Applied

Figure S9 shRNA targeting of *Npas2* and *Clock* successfully reduces expression in Control and REV-ERB DKO SCN slices. Related to Figure 5.

(A) *Npas2* expression for shCtrl-, shNpas2- and shNpas2 + shClock-treated Control (blue) and REV-ERB DKO SCN slices (orange) relative to Control + shCtrl.

(B) *Npas2* expression for Control (left, blue) and REV-ERB DKO (right, orange) SCN slices treated with shCtrl, shNpas2, or shNpas2 + shClock, relative to the shCtrl-treated mean for each group.

(C) *Clock* expression change for shCtrl-, shClock- and shNpas2 + shClock-treated Control (blue) and REV-ERB DKO SCN slices (orange) relative to Control + shCtrl.

(D) *Clock* expression for control (left, blue) and REV-ERB DKO (right, orange) SCN slices treated with shCtrl, shClock, or shClock + shNpas2, relative to the shCtrl-treated mean for each group. Gene expression calculated from RNA extracted from each slice from one of two trials included in **Figure 5**.

Figure S10

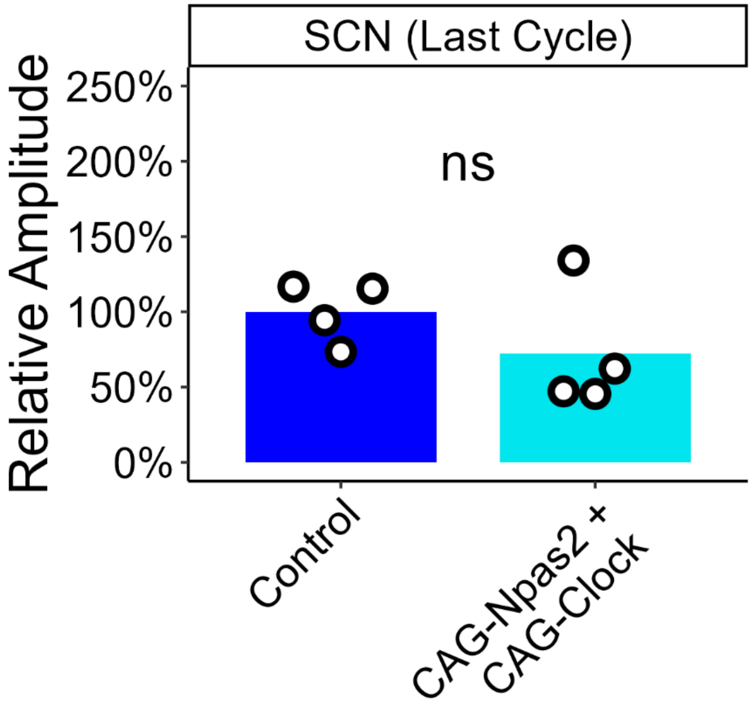

SCN (Last Cycle)
Relative Amplitude
250%
200%
150%
100%
50%
0%
ns
Control
CAG-Npas2 +
CAG-Clock

Figure S10. Simultaneous overexpression of *Npas2* and *Clock* does not impact the amplitude of SCN slices. Related to Figure 6. PER2::LUC amplitude of control (blue, mean ± SEM: 100 ± 10%, n = 4, 2 male, 2 female) and CAG-Npas2 + CAG-Clock-treated (light blue, mean ± SEM: 72 ± 21%, n = 4, 2 male, 2 female) SCN slices on the final cycle, relative to Control mean (*t*-test, p = 0.2791). Bar height represents mean value.
